# Distinct Signatures of Host Defense Suppression by Plant-Feeding Mites

**DOI:** 10.3390/ijms19103265

**Published:** 2018-10-20

**Authors:** Bernardus C. J. Schimmel, Juan M. Alba, Nicky Wybouw, Joris J. Glas, Tomas T. Meijer, Robert C. Schuurink, Merijn R. Kant

**Affiliations:** 1 Department of Evolutionary and Population Biology, Institute for Biodiversity and Ecosystem Dynamics, University of Amsterdam, P.O. Box 94240, 1090 GE Amsterdam, The Netherlands; bart.schimmel@ips.unibe.ch (B.C.J.S.); J.M.AlbaCano@uva.nl (J.M.A.); Nicky.Wybouw@UGent.be (N.W.); j.glas@rijkzwaan.nl (J.J.G.); tomastmeijer@hotmail.com (T.T.M.); 2 Department of Plants and Crops, Faculty of Bioscience Engineering, Ghent University, B-9000 Ghent, Belgium; 3 Department of Plant Physiology, Swammerdam Institute for Life Sciences, University of Amsterdam, P.O. Box 94215, 1090 GE Amsterdam, The Netherlands; R.C.Schuurink@uva.nl

**Keywords:** comparative transcriptomics, defense suppression, dual infestation, facilitation, herbivore, plant defense, plant-mediated interactions, tomato red spider mite (*Tetranychus evansi*), tomato russet mite (*Aculops lycopersici*), two-spotted spider mite (*Tetranychus urticae*)

## Abstract

Tomato plants are attacked by diverse herbivorous arthropods, including by cell-content-feeding mites, such as the extreme generalist *Tetranychus urticae* and specialists like *Tetranychus evansi* and *Aculops lycopersici*. Mite feeding induces plant defense responses that reduce mite performance. However, *T. evansi* and *A. lycopersici* suppress plant defenses via poorly understood mechanisms and, consequently, maintain a high performance on tomato. On a shared host, *T. urticae* can be facilitated by either of the specialist mites, likely due to the suppression of plant defenses. To better understand defense suppression and indirect plant-mediated interactions between herbivorous mites, we used gene-expression microarrays to analyze the transcriptomic changes in tomato after attack by either a single mite species (*T. urticae*, *T. evansi*, *A. lycopersici*) or two species simultaneously (*T. urticae* plus *T. evansi* or *T. urticae* plus *A. lycopersici*). Additionally, we assessed mite-induced changes in defense-associated phytohormones using LC-MS/MS. Compared to non-infested controls, jasmonates (JAs) and salicylate (SA) accumulated to higher amounts upon all mite-infestation treatments, but the response was attenuated after single infestations with defense-suppressors. Strikingly, whereas 8 to 10% of tomato genes were differentially expressed upon single infestations with *T. urticae* or *A. lycopersici*, respectively, only 0.1% was altered in *T. evansi*-infested plants. Transcriptome analysis of dual-infested leaves revealed that *A. lycopersici* primarily suppressed *T. urticae*-induced JA defenses, while *T. evansi* dampened *T. urticae*-triggered host responses on a transcriptome-wide scale. The latter suggests that *T. evansi* not solely down-regulates plant gene expression, but rather directs it back towards housekeeping levels. Our results provide valuable new insights into the mechanisms underlying host defense suppression and the plant-mediated facilitation of competing herbivores.

## 1. Introduction

Plants are usually attacked by multiple microbial and arthropod species, who attempt to consume them. To resists these attacks, plants have evolved a diverse set of constitutive and inducible defense traits [1,2]. The establishment and regulation of plant defenses critically depends on the action of two hormones: jasmonic acid (JA) and salicylic acid (SA). Whereas effective defenses against herbivores and necrotrophic pathogens generally require JA to accumulate, those against biotrophic pathogens depend on SA signaling [3,4]. The JA and SA signaling pathways interact, i.e., “crosstalk”, with each other as well as with signaling pathways of hormones that primarily regulate plant growth and development, presumably to fine-tune defense responses and to minimize growth-defense tradeoffs [5,6,7]. In turn, both herbivores and pathogens have evolved various traits that enable them to overcome plant defenses [8], for instance by suppressing them through exploitation of the host’s hormonal crosstalk mechanisms or through sabotage of the host’s molecular machinery [9,10].

By inducing or suppressing defenses, an attacking organism can indirectly influence the performance and behavior of other species that utilize the same host plant, e.g., species that either simultaneously or sequentially feed from a shared host, as well as their natural enemies [11,12,13]. Indirect plant-mediated interactions between phytophagous organisms are omnipresent and are major determinants of the plant-associated microbial and arthropod community composition (e.g., [14,15,16,17,18,19,20,21,22,23,24,25]). With respect to herbivorous arthropods, most plant-mediated interactions appear to be highly unidirectional and result in interference [18,26]. Although less frequent, plant-mediated interactions between herbivorous arthropods can also result in facilitation [18], for instance when one herbivore suppresses plant defenses or induces changes in the host’s resource availability which (also) benefit a second herbivore [27], potentially leading to reduced plant fitness and increased crop losses. Despite the ecological and agricultural relevance of plant-mediated interactions between herbivores, the underlying molecular mechanisms remain poorly understood [18,26,27,28,29].

Among the common pests of cultivated tomato (*Solanum lycopersicum*) are several species of mites (which are piercing-sucking, cell-content feeders), i.e., the generalist two-spotted spider mite (*Tetranychus urticae*) as well as the specialized tomato red spider mite (*Tetranychus evansi*) and the Eriophyid tomato russet mite (*Aculops lycopersici*) [23,30,31]. Unless one of these mite species manages to monopolize its host (e.g., via encapsulation with silken web [32]), they may indirectly interact with one another. This can have major consequences for population growth dynamics and, hence, may determine which species can develop into a pest and which not. For example, field-grown tomato plants that already suffered from a natural *A. lycopersici* infestation were frequently found to be invaded by *T. urticae* as well, while infestations in the reverse order hardly occurred [23]. Spider mite populations grew larger on plants with russet mites than on plants without [23]. Laboratory experiments demonstrated that *A. lycopersici* facilitated *T. urticae* on shared leaflets of intact tomato plants [23]. Additionally, *T. urticae* appeared to interfere with *A. lycopersici* population growth on such dual-infested leaflets, providing an explanation for the observed species-succession pattern [23]. Similarly, *T. urticae* can be facilitated by *T. evansi* when these mites share a tomato leaflet, i.e., either simultaneously [33] or sequentially [32], while *T. evansi*’s developmental rate, survival and reproductive performance all decrease dramatically when feeding on leaf material previously attacked by *T. urticae* as compared to on non-infested leaf material from control plants [30,32].

Exactly which plant processes or responses underlie these indirect interactions between mites is not known, but we hypothesize they largely result from plant defense suppression by *A. lycopersici* and *T. evansi*, respectively, in combination with defense induction by *T. urticae* [23,33]. This hypothesis is supported by several observations: Firstly, when feeding from tomato, *T. urticae* induces JA as well as SA-regulated defense responses that significantly reduce its performance (i.e., when not adapted to these defenses), with JA defenses being most important for resistance to *T. urticae* [34,35,36,37,38,39,40,41]. Accordingly, relative to wild-type (WT) plants, *T. urticae* performs much better on the JA biosynthesis mutant *defenseless-1* (*def-1*) [34,38]. In fact, the reproductive performance of this mite on *def-1* is as high as on WT leaflets that are simultaneously infested with *A. lycopersici* and, moreover, cannot be enhanced further via a co-infestation with russet mites [23]. This suggests that on WT plants *T. urticae* benefits from the suppression of JA defenses by *A. lycopersici*. Secondly, transcript levels of several JA-regulated, defense-associated marker genes (*Polyphenol Oxidase F*, *Threonine-Deaminase II*, *Jasmonate-Inducible Protein 21*, *Wound-Induced Proteinase Inhibitor II*) were significantly lower in tomato leaflets dual infested with *T. urticae* and *A. lycopersici* than in leaflets solely infested with *T. urticae*, but were still higher than or equal to the levels found in *A. lycopersici*-infested leaflets or in non-infested leaflets of control plants [23]. Note that expression of these genes was not induced upon single infestation with *A. lycopersici* [23]. The SA response showed the opposite pattern, i.e., an additive response, as the transcript level of the SA-regulated, defense-associated marker gene *Pathogenesis-Related Protein 6* was highest in dual-infested leaflets [23]. The concentrations of JA, JA-Ile (the main biologically active jasmonate) and SA were also highest in dual-infested leaflets [23]. In tomato plants carrying the *35S::nahG* transgene, which are unable to accumulate SA [42], the suppression of JA defense marker gene expression in dual-infested leaflets was no longer detected, whereas these genes were still not induced upon single infestation with russet mites. Together, this indicates that russet mites suppress JA defenses downstream from hormone accumulation and independently from JA-SA crosstalk [23]. Yet, the plant-mediated facilitation of *T. urticae* by *A. lycopersici* does depend on JA-SA crosstalk [23]. Thirdly, transcript levels of the JA defense marker gene *Proteinase Inhibitor IIc* and the SA defense marker gene *Pathogenesis-Related Protein 1a* were significantly lower in tomato leaflets dual infested with *T. urticae* and *T. evansi* than in leaflets solely infested with *T. urticae* [33]. Here too, expression of the marker genes was higher in the dual-infested leaflets than in leaflets solely infested with *T. evansi* or in non-infested leaflets of control plants [33]. This means that *T. evansi* suppresses both JA and SA defenses [33]. Like with russet mites, this suppression acts downstream from hormone accumulation and independently from JA-SA crosstalk [33]. It is important to point out that the suppression of gene expression in leaflets dual infested with inducer (*T. urticae*) and suppressor (*A. lycopersici* or *T. evansi*) mites was detected despite the presence of a higher number of mites on these leaflets, i.e., relative to the inducer mite-only treatment.

Although hormonal and especially transcriptomic changes in plants upon infestation with defense-inducing *T. urticae* have been well characterized in various species [33,37,39,41,43,44,45,46,47,48], our understanding of plant responses to suppressor mites is relatively limited [23,30,33,41,49,50,51,52]. Even less is known about how plants respond to a combined attack of defense-inducing and defense-suppressing mites, while previous analyses of such dual-infested plants have yielded valuable new insights into the mechanisms by which mites may facilitate competitors [23,33], as outlined earlier. Here, we have used LC-MS/MS-based methods and gene-expression microarrays to analyze phytohormonal and transcriptomic changes, respectively, in tomato leaflets after attack by either a single mite species (*T. urticae*, *T. evansi*, or *A. lycopersici*) or two species simultaneously (*T. urticae* plus *T. evansi*, or *T. urticae* plus *A. lycopersici*) in order to more rigorously test the hypothesis that plant-mediated interactions between these mites mainly result from the induction versus suppression of host defenses. We have exploited the specificity of defense induction by single and dual infestations to reveal the mechanisms and the outcome in terms of plant resistance. Given the importance of JA and SA for tomato resistance to mites [34,35,38,40,53], we have focused our transcriptome analysis on direct and indirect defense responses that are (predicted to be) regulated by these hormones. That is, we have surveyed relative expression levels of genes involved in the production of: antinutritive proteins, pathogenesis-related proteins, phenylpropanoid pathway-derived secondary metabolites, volatile organic compounds, and steroidal (glycol)alkaloids. Furthermore, we have investigated the possible involvement of crosstalk between JA/SA and other phytohormones during defense suppression. Finally, to find out if *T. evansi* and *A. lycopersici* target host processes other than defenses to trigger susceptibility, we have examined suppressor mite-specific effects on tomato gene expression.

We confirm that defense suppression takes place downstream from the accumulation of JA and SA, and show that it probably acts independently from hormonal crosstalk. Additionally, our results indicate that both specialist mites likely manipulate their host beyond the suppression of its defenses, as *T. urticae*-triggered tomato responses were dampened on a transcriptome-wide scale by *T. evansi* in dual-infested plants, and genes involved in cell cycle control and metabolite transport were specifically up-regulated in leaves (dual) infested with *A. lycopersici*. Nonetheless, our results also clearly demonstrate that suppression of both JA and SA-regulated defenses by *T. evansi* is distinct from the suppression of JA defenses by *A. lycopersici*.

## 2. Results

### 2.1. Defense-Associated Phytohormones

**Figure 1:**
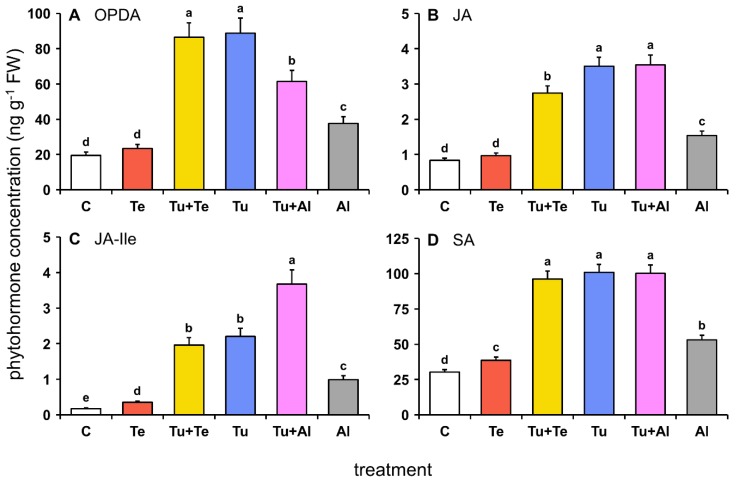
Phytohormone concentrations in tomato (*Solanum lycopersicum*) leaflets after seven days of infestation with herbivorous mites. Tomato leaves were infested with either a single mite species (*Tetranychus urticae* (Tu), *Tetranychus evansi* (Te), or *Aculops lycopersici* (Al)) or two species simultaneously (*T. urticae* plus *T. evansi* (Tu+Te), or *T. urticae* plus *A. lycopersici* (Tu+Al)). Non-infested plants served as controls (C). The figure shows the average (+ SEM) amounts of: (**A**) 12-oxo-phytodienoic acid (OPDA); (**B**) jasmonic acid (JA); (**C**) jasmonic acid-isoleucine (JA-Ile), and; (**D**) salicylic acid (SA). Multivariate analysis of variance indicated that the factor “mite-infestation treatment” had a significant effect on the phytohormonal profile (F_20_ = 8187; *p* < 0.001). Different letters above the bars indicate significant differences at a level of *p* ≤ 0.05, after applying a generalized linear model followed by Fisher’s Least Significant Difference test. Phytohormone concentrations are presented as nanogram per gram fresh leaf material (ng·g^−1^·FW).

Consistent with previous studies on tomato-mite interactions [23,33,54], higher concentrations of jasmonates and SA were detected after seven days of infestation with *T. urticae* (Tu) when compared to the non-infested control (C) plants (Figure 1). Note that mites from this *T. urticae* strain were originally collected from spindle tree (*Euonymus europaeus*), have subsequently been propagated on bean (*Phaseolus vulgaris*) for over ten years and, hence, are not adapted to tomato (see Section 4.2 Mites). Leaflets infested with *A. lycopersici* (Al) also contained higher amounts of defense-associated hormones, however the concentrations were consistently about two-fold lower than in the Tu samples. The infestation with *T. evansi* (Te) resulted in even smaller increases in the amounts of JA-Ile and SA, while no significant changes were detected for the JA-Ile precursors 12-oxo-phytodienoic acid (OPDA) and JA.

In general, the phytohormonal profiles of dual-infested leaflets were most similar to those of the Tu treatment (Figure 1). Yet, in leaflets infested with *T. urticae* plus *T. evansi* (Tu+Te), the JA concentration was intermediate as compared to that of each of the respective single infestations. Similarly, the amount of OPDA in leaflets infested with *T. urticae* plus *A. lycopersici* (Tu+Al) was intermediate as compared to OPDA amounts measured in the Tu and Al samples. By contrast, JA-Ile was most abundant in Tu+Al samples, indicative of an additive response.

### 2.2. Transcriptomic Profiles

Next, we isolated RNA from the same leaf tissue samples that were used for phytohormone extraction and performed gene-expression microarray analyses to get an overview of the transcriptomic responses of tomato plants upon single and dual mite infestations.

#### 2.2.1. General Overview of Mite-Induced Transcriptomic Changes in Tomato

**Figure 2:**
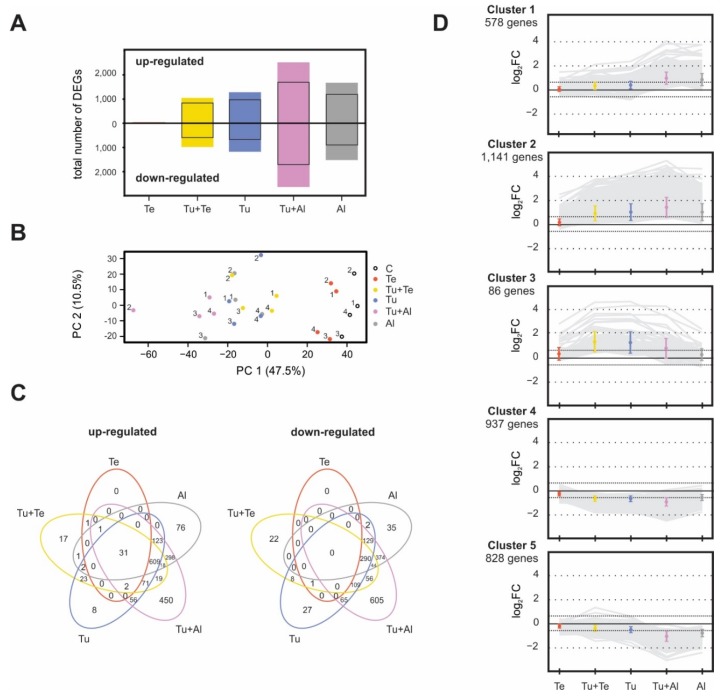
General overview of transcriptional responses in tomato (*Solanum lycopersicum*) leaves after seven days of infestation with herbivorous mites. Tomato leaves were infested with either a single mite species (*Tetranychus urticae* (Tu), *Tetranychus evansi* (Te), or *Aculops lycopersici* (Al)) or two species simultaneously (*T. urticae* plus *T. evansi* (Tu+Te), or *T. urticae* plus *A. lycopersici* (Tu+Al)). Non-infested plants served as controls (C) and were used as a common reference in the transcriptional comparisons. (**A**) The total numbers of differentially expressed genes (DEGs) across the five mite feeding regimes. Bars represent the number of up- or down-regulated tomato genes identified based on a Benjamini and Hochberg false discovery rate adjusted *p* ≤ 0.05. The black-lined sections within the bars indicate the number of up- or down-regulated tomato genes with an absolute fold change (FC) ≥ 1.5 (i.e., Log_2_FC ≥ 0.585). (**B**) Principal component analysis plot of the tomato transcriptomic responses to the five mite feeding regimes. (**C**) Venn-diagrams showing the overlap of the tomato transcriptomic responses to the five mite feeding regimes for up-regulated and down-regulated genes. (**D**) Transcriptional patterns of the five clusters of tomato DEGs across the five mite feeding regimes. Colored diamonds in each plot represent the average (±SD) of the transcript levels per feeding regime. The Log_2_FC cutoff value of 0.585 is depicted by dashed lines. The five clusters were identified using a *k*-means clustering approach.

We annotated our EST-based gene-expression microarray using the ITAG3.2 tomato genome annotation. The probes of our microarray platform represent 16,431 tomato genes, which corresponds to 53.2% of the 30,868 genes annotated in ITAG3.2. Using the C samples as a common reference, a total of 5494 differentially expressed genes (DEGs) were identified across all mite-infestation treatments at a Benjamini-Hochberg (BH) false discovery rate adjusted *p* ≤ 0.05. We found large variation in the number of DEGs in response to the different mite-infestation treatments (Figure 2A, Table S1). Most striking was the extremely low number of DEGs in the Te samples: only 38 were detected (0.1% of the genes annotated in ITAG3.2), from which all but one were up-regulated. In contrast, there were 2460 (8.0%) and 3200 (10.4%) DEGs in the Tu and Al samples, respectively, with an approximately equal number of genes being up- or down-regulated. For the dual-infested plants, we found 2032 DEGs (6.6%) in the Tu+Te samples, which is considerably less than in the Tu samples while leaflets were infested with twice as many mites. The opposite pattern was observed in the Tu+Al samples, which had as many as 5152 DEGs (16.7%). The number of up-regulated and down-regulated genes was similar in dual-infested plants.

For the subsequent transcriptomic analyses, we followed a more conservative approach by applying an additional cutoff at an absolute fold change (FC) of 1.5 (i.e., a Log_2_FC of 0.585), resulting in a total of 3570 DEGs across all mite-infestation treatments (Figure 2A, Table S1).

As a first step in our functional characterization of the mite-induced tomato transcriptomic responses, we performed a principal component analysis (PCA) based on the 3570 DEGs (absolute FC ≥ 1.5; BH-adjusted *p* ≤ 0.05). The PCA indicated that nearly half of the variance in differential gene expression could be attributed to the factor “mite-infestation treatment” (Figure 2B, PC 1). The Te and C samples clustered together but separated from the other infestation treatments, which is in line with the low number of DEGs between these treatments. Accordingly, the Tu+Al samples clustered furthest away from the controls along PC 1. The transcriptomic profiles of the Tu, Tu+Te, Al and Tu+Al samples did not separate clearly in the PCA plot (Figure 2B), which might be explained by the large overlap of DEGs across these treatments (Figure 2C). Thirty-one out of 35 genes up-regulated in *T. evansi*-infested leaflets were also up-regulated in response to each of the other mite infestation regimes. None of the genes on our microarray were found to be specifically (uniquely) up- or down-regulated by *T. evansi*, and only one (Solyc12g040860; encoding a glucan endo-1,3-β-glucosidase) was significantly up-regulated in the Te and Tu+Te samples but not in the others. With respect to *A. lycopersici*, a very different picture emerged from our transcriptome data analyses. Although the majority of up- and down-regulated Al-DEGs were shared with the other treatments, still 111 genes were specifically expressed in response to the *A. lycopersici* single infestation. Moreover, a total of 1836 genes (including those 111) were differentially regulated in the Al and/or Tu+Al samples but not in the others (Figure 2C).

As a second step in our functional characterization, the DEGs sets of each individual mite-infestation treatment were analyzed for enrichment of biological process (BP) gene ontology (GO) annotations. The Te treatment was excluded, because the number of Te-DEGs was insufficient for this analysis. Across the remaining infestation treatments, 21 BP GO categories were found to be significantly overrepresented in the tomato transcriptional responses, with substantial overlap between treatments (Table S2). Among the up-regulated DEGs in the Tu+Te, Tu, Tu+Al and Al samples, genes associated with the BP GO categories “recognition of pollen”, “protein phosphorylation”, “hydrogen peroxide catabolic process” and “chitin catabolic process” were significantly enriched (Table S2A), while down-regulated DEGs were consistently enriched in genes belonging to BP GO category “photosynthesis”. In addition, genes corresponding to the photosynthesis-related BP GO categories “photosynthesis, light harvesting” and/or “chlorophyll biosynthetic process” were overrepresented among down-regulated DEGs in the Tu, Tu+Al and Al samples (Table S2B).

Third, *k-*means clustering was performed on the 3570 DEGs based on the relative transcript levels across the mite-infestation treatments. Within this clustering analysis, the genes of clusters 1 and 2 were up-regulated in response to all mite-infestation treatments, except for Te, with those in cluster 1 being most strongly up-regulated in leaves (dual) infested with *A. lycopersici* (Figure 2D). The genes constituting clusters 4 and 5 were down-regulated in response to all mite-infestation treatments, except for Te, with those in cluster 5 being most strongly down-regulated in leaves (dual) infested with *A. lycopersici*. Whereas clusters 1 and 5 contained genes most strongly responding to *A. lycopersici*, the genes of cluster 3 were highly up-regulated in leaves (dual) infested with *T. urticae*, but much less so in response to single infestations with either *T. evansi* or *A. lycopersici*. Hence, cluster 3 is mainly comprised of Tu-specific DEGs. In each cluster, the mean FC was lowest for the Te treatment (Figure 2D), which may be explained by the low number of Te-DEGs. However, also for DEGs the magnitude of gene expression is lower in tomato leaves solely infested with *T. evansi* than in leaves (dual) infested with *T. urticae* or *A. lycopersici*. This is evident when comparing the 25 most highly up-regulated DEGs of the Tu, Te and Al samples (Tables S3–S5). Although, FC differences between the Tu+Te and Tu treatments were relatively small, the mean FC was smaller in the former in three out of five clusters. By contrast, the mean FC for the Tu+Al samples was higher than that of the Tu samples in four clusters (Figure 2D).

The DEGs belonging to each cluster were subsequently subjected to gene set enrichment analysis based on their BP GO annotations. This analysis revealed that cluster 3 (*T. urticae*-specific up-regulation) was significantly enriched in genes belonging to BP GO categories “response to wounding” and “pigment biosynthetic process”, whereas cluster 5 (strongest down-regulation in Tu+Al and Al samples) was enriched in genes belonging to BP GO categories “photosynthesis” and “photosynthesis, light harvesting”. The DEGs of the remaining clusters were not significantly enriched for any BP GO category.

#### 2.2.2. Detailed Analysis of Selected Defense-Associated Pathways

##### JA Pathway

**Figure 3:**
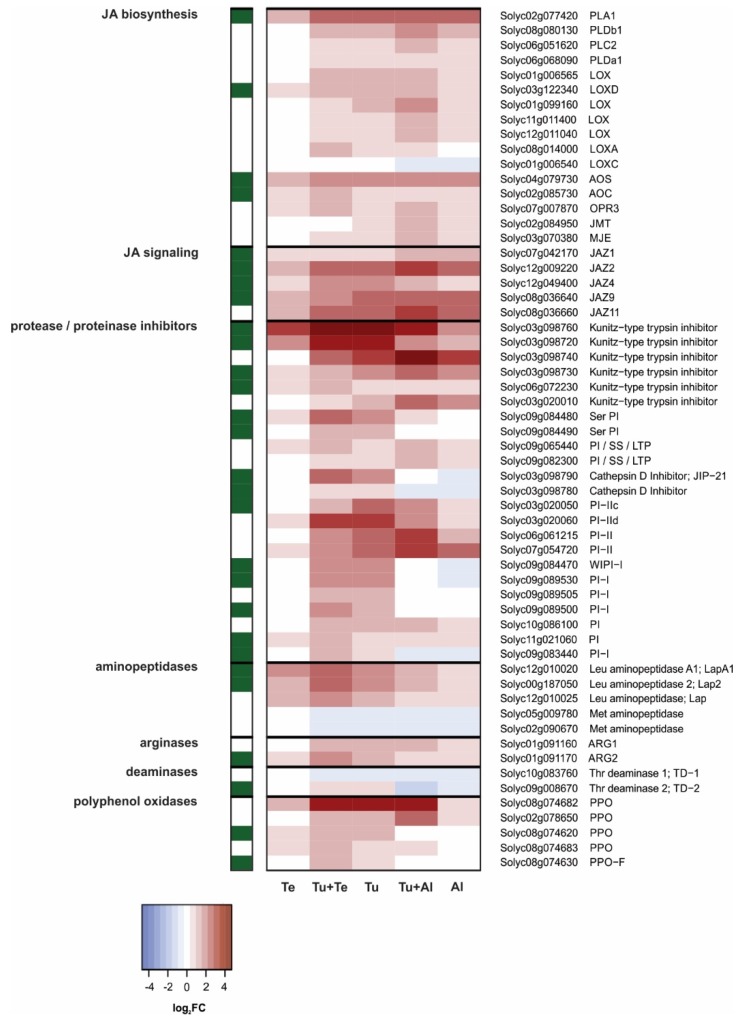
Gene-expression heat map depicting the relative transcript levels of tomato (*Solanum lycopersicum*) genes that encode proteins with a (predicted) function in the jasmonic acid (JA) pathway and that were differentially expressed in leaves after seven days of infestation with herbivorous mites. Tomato leaves were infested with either a single mite species (*Tetranychus urticae* (Tu), *Tetranychus evansi* (Te), or *Aculops lycopersici* (Al)) or two species simultaneously (*T. urticae* plus *T. evansi* (Tu+Te), or *T. urticae* plus *A. lycopersici* (Tu+Al). Non-infested plants served as controls and were used as a common reference in the transcriptional comparisons. Presented genes were differentially expressed (Benjamini and Hochberg false discovery rate adjusted *p* ≤ 0.05; Log_2_ fold change (FC) ≥ 0.585) in at least one of the mite-infestation treatments. The different (sub)sections of the pathway are specified on the left. Dark green squares in the leftmost column denote that transcription of the respective gene was found to be significantly induced in the tomato JA-biosynthesis mutant *def-1*, 24 h after exogenous application of JA (for details see [39]).

In general, jasmonate concentrations in mite-infested leaflets (Figure 1A–C) correlated well with both the detected number of DEGs with a predicted function in JA biosynthesis, metabolism or signaling as well as with the magnitude of their expression (Figure 3). Concurrent with the highly increased accumulation of jasmonates in leaflets (dual) infested with *T. urticae*, the majority of genes encoding (putative) JA biosynthesis enzymes [55], including phospholipases (PLs), lipoxygenases (LOXs), allene oxide cyclase (AOC), allene oxide synthase (AOC) and OPDA reductase 3 (OPR3), were significantly up-regulated in Tu+Te, Tu and Tu+Al samples (Figure 3). By contrast, the expression of just two JA biosynthesis genes (*PLA1* and *AOS*) was significantly up-regulated in *T. evansi*-infested leaflets. In the Al samples, *PL*s, *LOX*s and *AOS* were up-regulated, but expression of *AOC* was not significantly altered, while *OPR3* was induced, but less than 1.5-fold. In addition, a putative jasmonate methyltransferase (JMT)-encoding gene (Solyc02g084950) was significantly induced in Tu+Al and Al samples. Note that the gene coding for JAR1, i.e., the enzyme that catalyzes the conjugation of JA and Ile [56], is not present on our microarray. Immediately downstream from JA-Ile accumulation, i.e., at the JA-Ile perception and signaling level [55,57], multiple JASMONATE ZIM DOMAIN (JAZ)-encoding genes were induced upon each of the mite infestation treatments. *JAZ* induction, both in terms of gene numbers and magnitude of expression, was highest upon (dual) infestation with *T. urticae* and lowest with *T. evansi* (Figure 3). Expression of *COI1* and *MYC2* was not significantly altered in mite-infested plants.

Further downstream, concentrations of jasmonates still correlated well with the expression patterns of defense-associated genes in the Tu and Te samples, but no longer in the other samples, presumably due to suppression by *T. evansi* and *A. lycopersici*, respectively. In tomato, JA-induced responses to arthropod herbivores typically involve the production of various types of defensive proteins that have antinutritive properties [58], such as: proteinase/protease inhibitors (PIs) [59,60,61], arginases [62], threonine deaminases (TDs) [62,63], polyphenol oxidases (PPOs) [64], and possibly leucine aminopeptidases (Laps) [65,66]. These defensive proteins exert their antinutritive activity inside the herbivore’s digestive tract, thus after ingestion of plant material [58,67]. Multiple tomato genes encoding defensive antinutritive proteins of every class described above, except for TDs, were strongly up-regulated in the Tu samples (Figure 3). Four of them were among the 25 most highly up-regulated genes for the Tu treatment, including #1; Solyc03g098760, a PI (Table S3). This latter gene is also the top-ranking induced gene in the Te samples (Table S4), although the induction is much stronger in Tu (26-fold induction versus 7-fold). In total, four antinutritive protein-encoding genes were significantly up-regulated in Te (two PIs, one Lap, one PPO). Despite the high total number of up-regulated DEGs in Al samples, only seven of them encode putative JA-responsive antinutritive proteins (five PIs, one arginase, one PPO) and none of them were among the 25 most highly up-regulated Al-DEGs (Table S5). In addition, when directly compared with the antinutritive protein-encoding genes induced in Tu, the induction was considerably lower (i.e., absolute Log_2_FC difference > 0.2) for nearly 60% of them in Tu+Al, and for about 20% of them in Tu+Te, indicative of suppression by *A. lycopersici* and *T. evansi*, respectively. We verified this for several genes by means of qPCRs (Figure S1A–G). Our data indicates that suppressor mites do not suppress all antinutritive protein-encoding genes, i.e., at least at this single time point seven days after infestation. Furthermore, it is worth pointing out that in dual-infested leaves those genes suppressed by *A. lycopersici* were usually not suppressed by *T. evansi* and vice versa. For instance, the *T. urticae*-induced expression of Solyc03g098720, putatively coding for a Kunitz-type trypsin inhibitor (a PI), is suppressed by *A. lycopersici* but not by *T. evansi* in dual-infested plants (Figure S1C), while the opposite is observed for Solyc03g098740, which encodes another Kunitz-type trypsin inhibitor (Figure S1D).

To get an approximate idea of the extent to which the JA pathway genes depicted in Figure 3 are indeed JA-responsive, we cross-referenced our DEGs with genes whose expressions was significantly induced in *def-1*, 24 h after exogenous application of JA [39]. As expected, each section of the JA pathway contained JA-responsive genes and, except for *TD-2*, all were up-regulated in response to mite feeding, in particular in the Tu samples (Figure 3). Next, we applied custom filters to our microarray data in order to identify *A. lycopersici*-suppressed genes in dual-infested leaves (Table 1). We found that 23 out of the resulting 30 candidates were JA-inducible according to the data of Martel et al. [39]. To put this into perspective, applying the same filters to identify *T. evansi*-suppressed genes yielded a set of 31 candidates, four of which were JA-inducible (Table 2). Moreover, of the 25 most highly up-regulated DEGs, seven were JA-inducible for the Tu treatment (Table S3), 13 for the Te treatment (Table S4), yet zero for the Al treatment (Table S5). Our data, therefore, provides compelling evidence in support of the hypothesis that *A. lycopersici* predominantly suppresses JA-regulated defense responses [23].

##### SA Pathway

**Figure 4:**
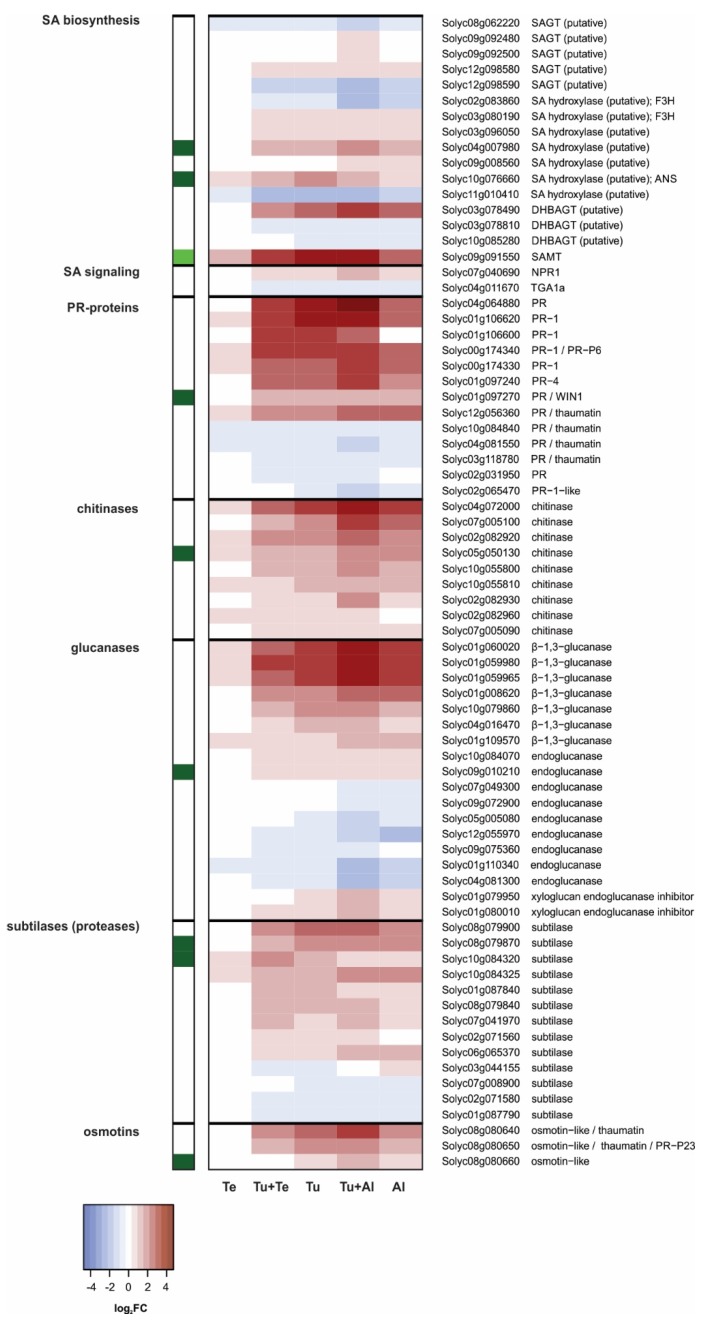
Gene-expression heat map depicting the relative transcript levels of tomato (*Solanum lycopersicum*) genes that encode proteins with a (predicted) function in the salicylic acid (SA) pathway and that were differentially expressed in leaves after seven days of infestation with herbivorous mites. Tomato leaves were infested with either a single mite species (*Tetranychus urticae* (Tu), *Tetranychus evansi* (Te), or *Aculops lycopersici* (Al)) or two species simultaneously (*T. urticae* plus *T. evansi* (Tu+Te), or *T. urticae* plus *A. lycopersici* (Tu+Al)). Non-infested plants served as controls and were used as a common reference in the transcriptional comparisons. Presented genes were differentially expressed (Benjamini and Hochberg false discovery rate adjusted *p* ≤ 0.05; Log_2_ fold change (FC) ≥ 0.585) in at least one of the mite-infestation treatments. The different (sub)sections of the pathway are specified on the left. Dark green squares in the leftmost column denote that transcription of the respective gene was found to be significantly induced in the tomato jasmonic acid (JA)-biosynthesis mutant *def-1*, 24 h after exogenous application of JA (for details see [39]). The light green square denotes JA-inducibility of the respective gene according to [75].

Despite the significantly increased concentrations of SA upon all mite infestation treatments, especially upon (dual) infestation with *T. urticae* (Figure 1D), changes in the relative expression level of genes predicted to be involved in SA biosynthesis, metabolism or signaling were relatively minor (Figure 4). The available gene expression data suggests that SA biosynthesis [68,69] in tomato upon infestation with mites proceeds via the phenylalanine ammonia lyase (PAL) pathway rather than via the isochorismate synthase (ICS) pathway. Tomato’s only *ICS* (Solyc06g071030) is not represented on our microarray platform, but we confirmed with qPCRs that it was not induced after seven days of feeding by any of our mite species (Figure S1H). In fact, *ICS* was down-regulated in *A. lycopersici*-infested leaves. Contrary to *ICS*, several early phenylpropanoid biosynthetic genes were induced by mite feeding (the phenylpropanoid pathway is discusses in more detail in the next section). One out of two chorismate mutase (CM)-encoding genes (Solyc02g088460) was significantly up-regulated in Tu+Te, Tu, Tu+Al and Al samples, but less than 1.5-fold. Genes coding for prephenate aminotransferase (PPA-AT; Solyc04g054710) and arogenate dehydratase (ADT; Solyc06g074530) enzymes were also up-regulated. The *PPA-AT* expression pattern was similar to that of *CM*, while *ADT* was induced to higher levels. Two *PAL* genes were significantly up-regulated in Tu samples, similar to the findings of Martel et al. [39], albeit only one (Solyc05g056170) by more than 1.5-fold. Expression of this latter *PAL* was induced to a similar level in Al, to slightly higher levels in Tu+Al, reduced to a level below the FC cutoff in Tu+Te, and was not significantly induced in Te. Despite their induction, the *CM*, *PPA-AT*, *ADT* and *PAL* expression patterns did not match the SA accumulation profiles very well, especially not in leaflets (dual) infested with *T. evansi*. This may be explained by the single and relatively late sampling moment. Additionally, it may be explained by active metabolism of SA. Expression patterns of tomato genes coding for putative homologs of *Arabidopsis thaliana* SA glycosyltransferases (SAGTs) [70,71], SA hydroxylases [72,73] and a dihydroxybenzoic acid glycosyltransferase (DHBAGT) [74], in particular those with the highest BLASTP scores, did not correlate well with SA levels. However, the expression pattern of *salicylic acid methyltransferase* (*SAMT*; Solyc09g091550) matched the SA accumulation profile much better (Figure 4). SAMT catalyzes the methylation of SA, generating volatile methyl salicylate (MeSA) in tomato plants [75,76], and may thereby have control over internal SA pools.

At the SA signaling level [68,77], expression of the tomato gene (Solyc07g040690) that encodes the putative homolog of *A. thaliana* NONEXPRESSOR OF PATHOGENESIS-RELATED GENES 1 (NPR1) was induced to similar levels in response to all mite-infestation treatments, except for the *T. evansi* infestation (Figure 4). A putative homolog of *A. thaliana* NPR3/4 (Solyc07g044980) had the same expression pattern. With regard to TGA transcription factors, we found one *TGA* gene to be down-regulated in Tu+Te, Tu, Tu+Al and Al, most strongly so in the latter two samples.

In sharp contrast with the SA biosynthesis, metabolism or signaling genes, we found massive changes in the expression of downstream defense-associated genes (Figure 4). Most SA-responsive genes associated with plant immunity code for so-called pathogenesis-related (PR) proteins that commonly have, or are predicted to have, antimicrobial and/or insecticidal properties [78]. The general picture that emerged from our microarray analysis of SA defense response marker genes is as follows: (i) The expression of dozens of genes encoding PR-proteins of various classes was highly induced in Tu and Al, often to the same extent, but was not induced in Te. (ii) Compared with the Tu and Al single infestations, in dual-infested leaves the number of up-regulated genes as well as the magnitude of their induction was higher in Tu+Al, while both factors were lower in Tu+Te (Figure 4). Using qPCRs, we verified these typical expression profiles for some SA marker genes (Figure S1I–J). Our results corroborate the report by Glas et al. [23] that *T. urticae* and *A. lycopersici* each induce SA-mediated defense responses and these are additive when mites from both species simultaneously infest the same leaflet. In line with the data of Alba et al. [33], our results also indicate that *T. evansi* suppresses SA defenses. In more detail: we found that for roughly 50% of the surveyed SA defense marker genes the magnitude of up-regulation was considerably lower (i.e., absolute Log_2_FC difference > 0.2) in Tu+Te than in Tu samples, with five of them being among the top-31 candidate *T. evansi*-suppressed genes (Table 2). This suppression was confirmed with qPCRs (Figure S1I–J).

##### Phenylpropanoid Pathway

**Figure 5:**
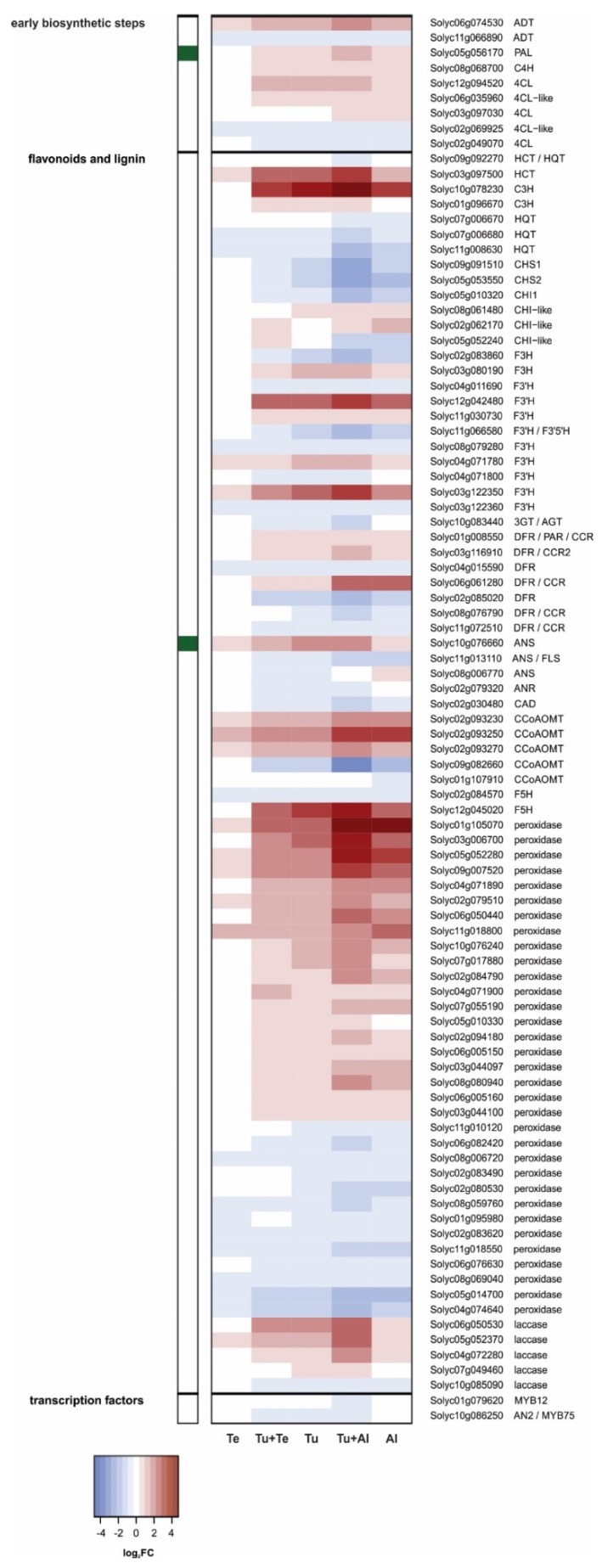
Gene-expression heat map depicting the relative transcript levels of tomato (*Solanum lycopersicum*) genes that encode proteins with a (predicted) function in the phenylpropanoid pathway and that were differentially expressed in leaves after seven days of infestation with herbivorous mites. Tomato leaves were infested with either a single mite species (*Tetranychus urticae* (Tu), *Tetranychus evansi* (Te), or *Aculops lycopersici* (Al)) or two species simultaneously (*T. urticae* plus *T. evansi* (Tu+Te), or *T. urticae* plus *A. lycopersici* (Tu+Al)). Non-infested plants served as controls and were used as a common reference in the transcriptional comparisons. Presented genes were differentially expressed (Benjamini and Hochberg false discovery rate adjusted *p* ≤ 0.05; Log_2_ fold change (FC) ≥ 0.585) in at least one of the mite-infestation treatments. The different (sub)sections of the pathway are specified on the left. Dark green squares in the leftmost column denote that transcription of the respective gene was found to be significantly induced in the tomato jasmonic acid (JA)-biosynthesis mutant *def-1*, 24 h after exogenous application of JA (for details see [39]).

Our transcriptional analysis of the phenylpropanoid metabolism in mite-infested leaflets yielded two main observations with potential biological relevance. The first observation is that, beside *CM* and *PAL*, genes putatively coding for the other core phenylpropanoid enzymes, i.e., cinnamate 4-hydroxylase (C4H) and 4-coumarate-CoA ligase (4CL) [79], were also induced upon (dual) infestation with *T. urticae* and *A lycopersici*, albeit not highly (Figure 5). This suggests that the production of one or more phenolic compounds increased in tomato plants upon these treatments. It proved difficult to pinpoint which compound(s), for the following four reasons: (i) Different isoforms exist of most phenylpropanoid enzymes, creating both substrate redundancy and specificity [79,80]. (ii) Phenylpropanoid enzymes do not always have strict substrate requirements and can therefore be involved in multiple branches of the pathway, generating various products [79,80]. (iii) Genes (putatively) coding for functionally equivalent enzymes had opposite expression patterns in the same sample. (iv) Genes (putatively) coding for enzymes that sequentially function in the same biosynthetic pathway had opposite expression patterns. For instance, genes predicted to encode enzymes that: (a) convert naringenin into various dihydroflavonols (flavone-3-hydroxylase [F3H], flavonoid 3’-hydroxylase [F3’H], flavonoid 3’,5’-hydroxylase [F3’5’H]); (b) catalyze the first dedicated reaction towards the production of flavonols from dihydroflavonols (flavonol synthase [FLS]), and/or; (c) catalyze the first two committed steps of anthocyanin production from dihydroflavonols (dihydroflavonol reductase [DFR] and anthocyanidin synthase [ANS], respectively), had inconsistent expression patterns (Figure 5). Nonetheless, the production of the phenylpropanoid chlorogenic acid (CGA) may be promoted upon (dual) infestation with *A. lycopersici*. CGA is one of the most abundant phenolics in tomato leaves [81,82,83] and has been suggested to confer antinutritive properties in the herbivore’s gut, i.e., upon oxidation by e.g., co-ingested PPOs [84,85,86]. In tomato, the synthesis of CGA requires hydroxycinnamoyl-CoA quinate hydroxycinnamoyl transferase (HQT), ρ-coumarate 3’-hydroxylase (C3H), and possibly hydroxycinnamoyl-CoA transferase (HCT) activity [87]. Expression of the gene encoding key enzyme HQT (Solyc07g005760; [87]) was significantly induced in Tu+Al and Al samples, but less than 1.5-fold. Putative HCT and C3H-encoding genes were highly induced in response to all mite infestations, except for the *T. evansi* single infestation (Figure 5).

The second observation is that tomato’s two established chalcone synthase (CHS)-encoding genes, *CHS1* and *CHS2* [88,89], as well as one of its two canonical chalcone isomerase (CHI)-encoding genes, *CHI1* [90], were down-regulated in the Tu, Tu+Al and Al samples, most strongly so in Tu+Al (Figure 5). Note that *CHI2* is normally not expressed in leaves [90]. *CHS1*, *CHS2* and *CHI1* appeared to be down-regulated in Tu+Te samples as well, but this was only statistically significant for *CHI1*. No such changes were observed in Te. By means of qPCRs, we confirmed the expression patterns of *CHS1* and *CHS2* (Figure S2A,B). We additionally found that several genes encoding positive regulators of flavonoid (MYB12; [91,92,93,94]) or anthocyanin biosynthesis (AN2/MYB75, JAF13/bHLH90; [95]) were also down-regulated in Tu+Al. The anthocyanin biosynthesis regulatory gene *ANT1/MYB113* [96] was not differentially regulated in response to mite feeding. Together, our results suggest that the production of flavonoids and anthocyanins may be decreased in leaflets (dual) infested with *T. urticae* and/or *A. lycopersici*.

We assessed whether genes associated with lignin biosynthesis were up-regulated in Tu+Te, Tu, Tu+Al and Al, as an altered flux through the phenylpropanoid pathway [79] may explain the apparent down-regulation of flavonoid/anthocyanin biosynthesis in these samples. Our results are inconclusive, because we found up-regulated as well as down-regulated lignin-biosynthesis genes in the same samples (Figure 5). Most relevant in this respect is that the genes coding for key enzymes cinnamoyl-CoA reductase (CCR) and cinnamyl alcohol dehydrogenase (CAD) had contrasting expression patterns. Of the two *bona fide* CCR-encoding genes, *CCR1* (Solyc06g068440) and *CCR2* (Solyc03g116910) [81], only the latter was differentially regulated. *CCR2* expression was approximately 2-fold higher in Tu+Al than in C samples. No significant changes were detected in the other samples. The only DEG among putative CAD-encoding genes, Solyc02g030480, was down-regulated in Tu+Al, as well as in the Tu and Al single infestation samples. Lastly, we detected dozens of differentially regulated peroxidase and laccase-encoding genes in Tu+Te, Tu, Tu+Al and Al, but none in Te. The majority of these genes were up-regulated (Figure 5). Hence, lignin polymerization activity may be increased in response to *T. urticae* and *A. lycopersici* feeding.

##### Terpenoids

**Figure 6:**
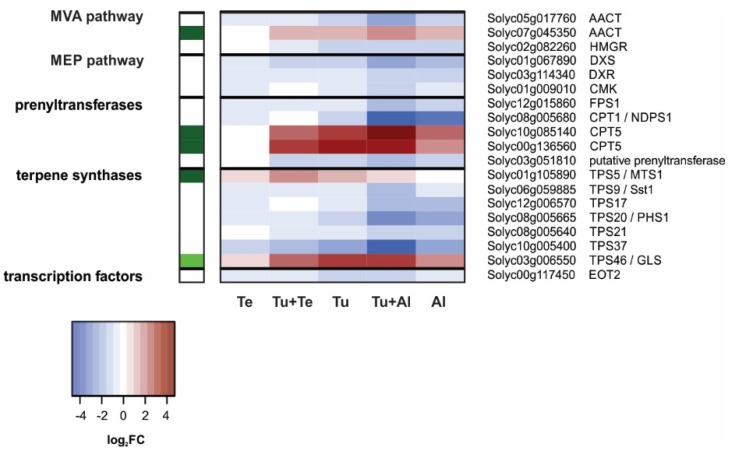
Gene-expression heat map depicting the relative transcript levels of tomato (*Solanum lycopersicum*) genes that encode proteins with a (predicted) function in terpenoid biosynthesis and that were differentially expressed in leaves after seven days of infestation with herbivorous mites. Tomato leaves were infested with either a single mite species (*Tetranychus urticae* (Tu), *Tetranychus evansi* (Te), or *Aculops lycopersici* (Al)) or two species simultaneously (*T. urticae* plus *T. evansi* (Tu+Te), or *T. urticae* plus *A. lycopersici* (Tu+Al)). Non-infested plants served as controls and were used as a common reference in the transcriptional comparisons. Presented genes were differentially expressed (Benjamini and Hochberg false discovery rate adjusted *p* ≤ 0.05; Log_2_ fold change (FC) ≥ 0.585) in at least one of the mite-infestation treatments. The different (sub)sections of the pathway are specified on the left. Dark green squares in the leftmost column denote that transcription of the respective gene was found to be significantly induced in the tomato jasmonic acid (JA)-biosynthesis mutant *def-1*, 24 h after exogenous application of JA (for details see [39]). The light green square denotes JA-inducibility of the respective gene according to [97].

Overall, transcriptional changes in the terpenoid biosynthetic pathway of our mite-infested tomato plants were relatively minor in terms of both the number of DEGs as well as their FCs (Figure 6). Most terpenoid-DEGs and highest FCs were observed in the Tu+Al samples, while no significant changes were found in Te. Here, we highlight four main findings of our transcriptional analysis of the terpenoid pathway. 

Firstly, we noted the strong, simultaneous down-regulation of *CPT1/NDPS1* and *TPS20/PHS1* in tomato leaves (dual) infested with *A. lycopersici* (Figure 6). These genes appeared to be down-regulated in Tu samples as well, but this was not statistically significant. *CPT1/NDPS1* is one of the most strongly down-regulated genes in the Al samples (Table S6; #3) and encodes a neryl diphosphate (NPP)-producing cis-prenyltransferase [98]. The terpene synthase (TPS) encoded by *TPS20/PHS1* catalyzes the conversion of NPP into several monoterpenes (C_10_), including α- and β-phellandrene [98]. The highly reduced expression of *CPT1/NDPS1* and *TPS20/PHS1* in *A. lycopersici*-infested leaves suggests that phellandrene may be emitted in lower amounts by these plants as compared to non-infested controls. Although the magnitude of FCs was smaller than for *CPT1/NDPS1* and *TPS20/PHS1*, we found similar expression patterns for *TPS17* and *TPS21* (Figure 6). *TPS17* encodes a sesquiterpene (C_15_) synthase [99] and *TPS21* a diterpene (C_20_) synthase [100]. The main reaction products of these enzymes are valencene and lycosantalene, respectively [99,100].

Secondly, consistent with the increased emission of the monoterpene linalool, the sesquiterpene (*E*)-nerolidol and the homoterpene (C_16_) (*E*, *E*)-4,8,12-trimethyltrideca-1,3,7,11-tetraene (TMTT) in response to *T. urticae* feeding [37], expression of two TPS genes involved in the biosynthesis of these compounds, i.e., *TPS5/MTS1* and *TPS46/GLS*, was significantly up-regulated in Tu samples. The trichome-localized TPS5/MTS1 preferentially produces linalool from geranyl diphosphate (GPP), yet can also generate (*E*)-nerolidol from farnesyl diphosphate (FPP) [101]. *TPS5/MTS1* was induced in Tu+Te as well, but not in leaflets (dual) infested with *A. lycopersici*. TPS46/GLS, uses geranylgeranyl diphosphate (GGPP) as a substrate to produce the diterpene geranyllinalool [97], which is the precursor of TMTT. The enzyme(s) responsible for the conversion of geranyllinalool into TMTT has/have not been identified in tomato. Although enzymatically less efficient, TPS46/GLS can synthesize (*E*)-nerolidol from FPP [97]. *TPS46/GLS* expression was up-regulated upon all mite infestation treatments, except for the single infestation with *T. evansi*. It should be noted that whereas *TPS5/MTS1* was induced by *T. urticae* feeding, *TPS37* was down-regulated (also in Tu+Te, Tu+Al and Al samples). This is noteworthy because TPS5/MTS1 and TPS37 are functionally equivalent [102]. However, *TPS5/MTS1* transcripts seem to be far more abundant than those of *TPS37*, especially in glandular trichomes of JA-treated plants [102].

Thirdly, genes encoding the prenyltransferases responsible for the production of GPP (i.e., GPS [103]), FPP (i.e., FPSs [104]) or GGPP (i.e., GGPSs [105]) were not up-regulated by mite feeding. One exception may be *GGPS1*, whose expression is known to be induced by *T. urticae* [105] and suppressed by *T. evansi* [30], as this gene is not present on our microarray. Another prenyltransferase gene, *CPT5*, which encodes a polyisoprenyl diphosphate (C_55_–C_80_)-generating enzyme [106] was induced in all samples, except for Te (Figure 6). CPT5 uses FPP as substrate [106], yet *FPS1* [104] was down-regulated in samples where *CPT5* was induced. The function of polyisoprenoids in plant-mite interactions is unknown.

Fourthly, further upstream in the terpenoid biosynthetic pathway, several genes coding for enzymes belonging to the cytosolic mevalonate (MVA) pathway (acetoacetyl-CoA thiolase [AACT]; 3-hydroxy-3-methylglutaryl-CoA reductase [HMGR]) or the plastidial 2-C-methyl-d-erythritol 4-phosphate (MEP) pathway (1-deoxy-d-xylulose 5-phosphate synthase [DXS]; 1-deoxy-d-xylulose 5-phosphate reductoisomerase [DXR]; 4-diphosphocytidyl-2-C-methyl-d-erythritol kinase [CMK]) were down-regulated in leaves (dual) infested with *T. urticae* and/or *A. lycopersici* (Figure 6).

##### Steroidal (glyco)alkaloids

Solanaceous plants typically produce steroidal alkaloids. These nitrogen-containing secondary metabolites are commonly glycosylated, presumably to reduce their autotoxicity, forming steroidal glycoalkaloids (SGAs) [107,108]. Steroidal (glyco)alkaloids are generated from cholesterol [108] (which in turn is produced from the triterpene (C_30_) squalene [109]) via a series of hydroxylation, oxidation, transamination, reduction and/or glycosylation reactions that are catalyzed by various GLYCOALKALOID METABOLISM (GAME) enzymes, of which ten have been characterized in tomato thus far [107,108,110,111]. In addition, the JA-responsive transcription factor GAME9/JRE4 functions as a master regulator of steroidal (glyco)alkaloid metabolism by controlling, either alone or in association with MYC2, the expression of genes coding for key enzymes in the MVA, cholesterol and SGA biosynthesis pathways [112,113,114].

**Figure 7:**
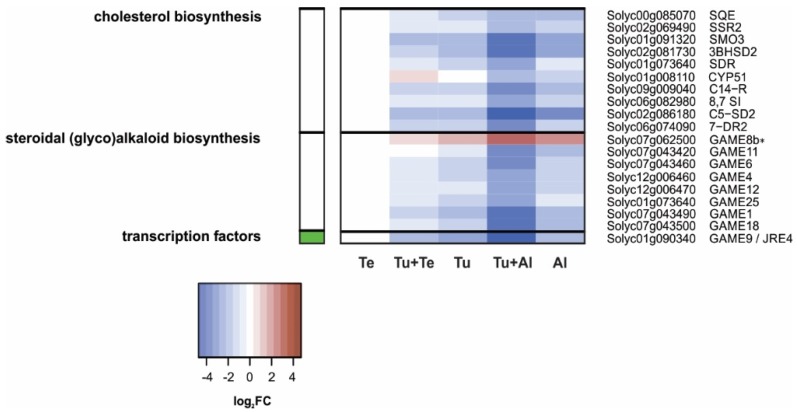
Gene-expression heat map depicting the relative transcript levels of tomato (*Solanum lycopersicum*) genes that encode proteins with a (predicted) function in cholesterol and steroidal (glyco)alkaloid biosynthesis and that were differentially expressed in leaves after seven days of infestation with herbivorous mites. Tomato leaves were infested with either a single mite species (*Tetranychus urticae* (Tu), *Tetranychus evansi* (Te), or *Aculops lycopersici* (Al)) or two species simultaneously (*T. urticae* plus *T. evansi* (Tu+Te), or *T. urticae* plus *A. lycopersici* (Tu+Al)). Non-infested plants served as controls and were used as a common reference in the transcriptional comparisons. Presented genes were differentially expressed (Benjamini and Hochberg false discovery rate adjusted *p* ≤ 0.05; Log_2_ fold change (FC) ≥ 0.585) in at least one of the mite-infestation treatments. The different (sub)sections of the pathway are specified on the left. The light green square in the leftmost column denotes JA-inducibility of the respective gene according to [114]. The asterisk indicates that the enzyme encoded by this GAME gene has not been characterized in tomato, i.e., only in potato [115].

We observed that expression of eight out of nine *GAME* genes present on our microarray was down-regulated in tomato leaves dual infested with *T. urticae* and *A. lycopersici* (Figure 7). Although the FCs were smaller, the same genes were down-regulated upon single infestation with *A. lycopersici*, for six of them this down-regulation was statistically significant. *GAME9*/*JRE4* was the most strongly down-regulated *GAME* gene in both Tu+Al and Al samples, followed by *GAME1* and *GAME18*. The latter two code for glycosyltransferases that are responsible for two out of four sugar-attachment steps required for the conversion of tomatidine into α-tomatine, which is the major SGA present in green tomato tissues [107,108]. The genes encoding the other two glycosyltransferases, *GAME2* and *GAME17* [108], are not present on our microarray. Expression of some *GAME* genes appeared to be down-regulated in the Tu samples, but this was only statistically significant for *GAME9*/*JRE4*. No changes were found in the Te samples. We verified the expression patterns of *GAME1* and *GAME9/JRE4* with qPCRs (Figure S2C,D). Very similar expression patterns were found for the putative cholesterol biosynthesis genes [109] (Figure 7) as well as for genes encoding enzymes of the upstream MVA pathway, as discussed earlier (Figure 6). Our results suggest that the steroidal (glyco)alkaloid contents of tomato leaves may be reduced and the sterol composition altered upon infestation with *A. lycopersici*, possibly with *T. urticae* as well, and that those changes are most severe when mites from both species simultaneously attack the plant.

#### 2.2.3. Hormonal Crosstalk

To gather more insight into whether or not *T. evansi* and/or *A. lycopersici* suppress plant defenses by exploiting crosstalk mechanisms with other phytohormones, we surveyed expression profiles of DEGs (putatively) involved in the biosynthesis of- and signaling by abscisic acid, auxin, brassinosteroids, cytokinins, ethylene and gibberellins (Figure S3). In short, there was no clear indication for crosstalk between any of these hormones and the JA or SA pathway, for four main reasons. (i) Most DEGs associated with hormones that primarily regulate growth and development (“G&D”) were regulated similarly in plants infested with defense-inducing *T. urticae* versus in plants infested with defense-suppressing *T. evansi* or *A. lycopersici*. Only one G&D hormone-DEG (Solyc01g107400, a putative IAA-amido synthetase) had a contrasting expression pattern; it was up-regulated in Al, but down-regulated in Tu. (ii) Each G&D hormone pathway consisted of a mixture of up and down-regulated DEGs. No pathway was uniformly regulated in response to any of the mite-infestation treatments. (iii) The magnitude of FCs was lower for G&D hormone-DEGs than for DEGs of the JA and SA pathways. Several of the most highly up-regulated (putative) G&D hormone-DEGs, including Solyc01g107390 (auxin-responsive *GH3*), Solyc04g080820 (*cytokinin oxidase 4*) and Solyc07g049530 (ethylene biosynthesis gene *ACC oxidase*), are actually also responsive to JA [39]. (iv) G&D hormone pathways, or subsections thereof, did not surface in our enrichment analyses. In Tu+Te samples, the magnitude of FCs of various G&D hormone-DEGs was reduced as compared to their expression in Tu. This was observed for up as well as down-regulated genes in all hormone pathways. The opposite expression pattern (an additive response) was apparent when Tu+Al samples were compared with Tu (Figure S3).

#### 2.2.4. Beyond Suppression of *T. urticae*-Induced Genes

Numerous DEGs were exclusively found in leaves (dual) infested with *A. lycopersici* (Figure 2C). Given that these genes were not differentially regulated in Tu samples, they may provide insight into the mechanism(s) underlying defense suppression by *A. lycopersici*. We therefore mined the microarray data for those genes that were up-regulated in the Al samples but were not differentially expressed (BH-adjusted *p* > 0.05) in the Tu ones. The resulting 612 genes (the top 25 is presented in Table S7) appeared to be involved in various processes, but among the genes with the largest absolute difference in expression level in Al versus Tu were genes putatively involved in cell wall organization (e.g., expansins, pectinesterases, peroxidases), cell cycle control (e.g., cyclins), transport (e.g., of amino acids, ammonium, sugars) and defense/secondary metabolism (e.g., cytochrome P450’s, GDSL esterases/lipases, β-1,3-glucanases). Very few of these genes were JA-responsive according to the data of Martel et al. [39]. Next, we searched the microarray data for genes that were down-regulated in the Al samples but were not differentially regulated in Tu samples. This yielded a set of 715 genes (the top 25 is presented in Table S8), many of these appeared to be involved in photosynthesis (e.g., subunits of photosystem I or II, chlorophyll A-B binding proteins, thylakoid proteins), which is in line with the gene set enrichment analysis (Table S2B). Furthermore, several of the Al (and Tu+Al)-specific DEGs coded for hormone-responsive proteins, but genes encoding proteins that were responsive to the same hormone were present in the lists of up-regulated as well as down-regulated genes, as discussed in the previous section.

Compared with the non-infested controls, there were 1179 tomato genes whose expression was significantly down-regulated in *T. urticae*-infested leaves (Table S1). In total 401 of these genes, i.e., 34%, were no longer down-regulated in leaves dual infested with *T. urticae* and *T. evansi*. Among them are some of the genes that are strongly down-regulated in the Tu samples (Table S9) and/or that may be linked to plant defense/susceptibility, such as: putative pattern-recognition receptors (PRRs: Solyc11g056680, Solyc01g107670), CHS1, CHS2, a sucrose synthase (Solyc07g042520) and pectate lyases (Solyc05g014000, Solyc06g083580). As we were unable to verify this gene expression pattern for *CHS1* and *CHS2* by means of qPCRs (Figure S2A,B), this result may in part be explained by the statistical methods employed to analyze the transcriptomic data. However, the number of genes that is no longer down-regulated in Tu+Te versus Tu seems disproportionally large with respect to the absolute difference in down-regulated genes between these samples, which is 196 genes (Table S1). We did not find anything similar for the Tu+Al versus Tu comparison, in which 72 of the 1179 genes (6.1%) were no longer down-regulated in leaves dual infested with *T. urticae* and *A. lycopersici* as compared to in *T. urticae*-infested leaves. The total number of down-regulated genes is much larger for the Tu+Al samples than for the Tu+Te ones (Figure 2A; Table S1). These results suggest that *T. evansi* not only suppresses induced defenses, but also counteracts the down-regulation of plant responses triggered by *T. urticae*.

## 3. Discussion

In this study, we have analyzed the phytohormonal and transcriptomic changes in tomato plants upon single or dual (simultaneous) infestations with naturally competing species of mites that differ in their ability to induce or suppress host defenses. We show that single infestations with specialist, defense-suppressing *A. lycopersici* or *T. evansi* both triggered the increased accumulation of JA-Ile and SA, yet yielded very different transcriptomic changes in tomato leaves. The latter is likely the result of distinct suppression mechanisms employed by these mites. Whereas *A. lycopersici* predominantly suppressed JA-regulated direct and indirect defense responses, *T. evansi* suppressed both JA and SA-regulated defenses. Moreover, we provide evidence that both specialist mites probably (also) manipulated host processes other than immune responses.

The finding that JA-Ile and SA accumulated to similar or higher amounts in dual-infested leaflets as compared to leaflets infested with *T. urticae* alone (Figure 1) is in agreement with results from previous studies [23,33]. It implies that suppression of host defenses by *A. lycopersici* and *T. evansi* acts downstream from phytohormone accumulation and independently from JA-SA crosstalk. Indeed, there were no indications that these mites actively suppressed JA and/or SA biosynthesis genes (Figure 3 and Figure 4). One exception may be the suppression of two PAL-encoding genes by *T. evansi*, but our evidence for this is weak. In addition, at the transcriptome level there were no indications that suppressor mites exploited crosstalk between hormones that are critical for plant immunity (i.e., JA, SA) versus those that primarily regulate growth and development (i.e., abscisic acid, auxin, brassinosteroids, cytokinins, ethylene, gibberellins). If not via the suppression of biosynthetic genes or crosstalk with other hormones, russet mites may nonetheless limit the accumulation of JA-Ile via the induction of a *jasmonate methyltransferase* (*JMT*: Figure 3). In wild tobacco (*Nicotiana attenuata*) and rice (*Oryza sativa*), elevated *JMT* expression dampens herbivory-induced JA-Ile accumulation by redirecting the JA flux towards production of methylated JA (MeJA), thereby improving herbivore performance [116,117,118].

Clustering and enrichment analyses of the transcriptomic changes in tomato in response to feeding by *T. urticae* and/or *A. lycopersici* revealed gene expression activity associated with several core biological processes of plant immunity, that is: (i) perception of herbivore attack; (ii) signaling events immediately downstream from this perception; (iii) production of anti-herbivore compounds, such as defensive proteins and secondary metabolites, and; (iv) inhibition of photosynthesis [2,119]. In this respect, the enrichment of genes belonging to BP GO category “recognition of pollen” among the up-regulated DEGs in mite-infested leaves (except for in Te samples) is noteworthy. Several of the genes of this category encode putative lectin-type PRRs. The recognition of (damaged) self versus non-self is a crucial aspect of plant immunity as well as of reproduction and can be governed by lectin-type PRRs [120,121,122]. PRRs required for plant resistance to mites remain to be identified. As ligand binding by a PRR often induces the expression of the corresponding PRR-encoding gene [123], the increased expression of several lectin-type PRR genes further supports our hypothesis that they may be involved in the recognition of mite attack.

The highest number of DEGs (5132) was found in leaves dual infested with *T. urticae* and *A. lycopersici*, i.e., in leaves with the highest density of mites. The plant’s response to this dual attack was mostly additive for up- as well as down-regulated genes. The additive response was evident, among others, for the induced SA defenses, while the JA defenses formed a clear exception. The *T. urticae*-induced up-regulation of canonical JA-responsive genes that code for antinutritive proteins (e.g., proteinase inhibitors, aminopeptidases, polyphenol oxidases) was dramatically reduced or fully compromised in Tu+Al samples (Figure 3 and Figure S1; Table 1). We observed the same expression pattern for the JA-responsive *TPS5/MTS1*, which encodes the terpene synthase responsible for linalool production [101]. These results provide clear evidence for the suppression of JA defenses by russet mites. Note that this suppression is detected despite the much higher number of mites on the dual-infested plants, i.e., compared to the single infestation with *T. urticae*. Accordingly, expression of most of the JA-responsive defense-associated genes was not induced upon single infestation with *A. lycopersici*. We additionally found that the expression levels of several genes associated with other (potential) defense responses were down-regulated in plants (dual) infested with *A. lycopersici*, i.e., well below levels found in non-infested control plants. This concerns the genes coding for: (a) CHS1, CHS2 and CHI1 of the phenylpropanoid pathway (Figure 5), i.e., enzymes that fulfill gatekeeper functions in flavonoid and anthocyanin biosynthesis [79,88,89,90]; (b) CPT1/NDPS1 and TPS20/PHS1 (Figure 6), which are responsible for the production of the volatile monoterpene phellandrene in glandular trichomes [98], and; (c) GAME enzymes as well as the main JA-regulated GAME transcription factor (Figure 7), which together are required for the production of steroidal (glycol)alkaloids [108,113]. Hence, the production of various classes of secondary metabolites (flavonoids, anthocyanins, terpenes, steroidal glycoalkaloids) may be attenuated in leaves (dual) infested with *A. lycopersici*. This is reminiscent of a recent report on whitefly (*Bemisia tabaci*)-infested tomato plants, whose leaves contained lower amounts of various flavonoids and anthocyanins, while also the emission of α-phellandrene from these plants was reduced [124]. Whiteflies have a higher fitness on tomato leaves that have previously been infested by conspecifics than on leaves of non-infested control plants and strongly prefer to feed and oviposit on such conspecific-infested leaves [124]. The lower flavonoid levels in infested leaves were found to be causally linked with *B. tabaci*’s oviposition preference, whereas the reduced α-phellandrene emission was causally linked with *B. tabaci*’s foraging preference for conspecific-infested plants [124]. Interestingly, the decreased flavonoid and α-phellandrene production coincided with reduced transcript levels of tomato *CHS1*, *CHI1*, *FLS*, *DFR* and *TPS20/PHS1* [124]. It was therefore suggested that whiteflies suppress the accumulation of specific flavonoids, anthocyanins and terpenoids to alter the behavior of conspecifics, which may ultimately enhance their performance [124]. Whether the here detected down-regulation of *CHS1*, *CHS2*, *CHI1*, *CPT1/NDPS1* and *TPS20/PHS1* (as well as the *GAME* genes) has a similar function in russet mite infestations and, hence, is herbivore-adaptive, remains unclear. For instance, because russet mites are less mobile than whitefly adults and because the down-regulation of trichome-localized terpenoid biosynthesis genes may (also) be associated with the deterioration of glandular trichomes, a phenotype commonly observed on russet mite-attacked plants [125]. Finally, although the analysis of Al (and Tu+Al)-specific DEGs did not provide clear information on how *A. lycopersici* suppresses plant defenses, we did note the specific up-regulation of genes involved in cell cycle control or in the transport of various metabolites. Whereas spider mites feed from mesophyll cells whilst avoiding to pierce the epidermis [126], russet mites are much smaller and therefore restricted to feed from epidermal cells. Since the content of normal epidermal cells is presumably not very nutritious for mites, *A. lycopersici* may manipulate ploidy levels (e.g., of underlying mesophyll cells) and redirect the transport of nutrients in infested leaves to meet its metabolic demands, similar to what some plant-feeding microbes do [127,128,129]. This suggests that russet mites may manipulate more than just defense responses to increase the susceptibility of their host and, thus, to promote their own proliferation. 

Much to our own surprise, we found only 38 tomato genes to be differentially regulated after seven days of infestation with *T. evansi*, as compared to the 3200 and 2460 DEGs upon single infestations with *A. lycopersici* or *T. urticae*, respectively. This is extra remarkable given that *T. evansi* is known to cause ca. 2-fold more feeding damage on tomato than *T. urticae* [33]. This means that the low number of DEGs in the Te samples must be the result of host plant manipulation by *T. evansi*, rather than of limited feeding. As with *A. lycopersici*, our current analysis did not reveal the exact mechanism(s) by which *T. evansi* suppresses plant responses. The main reason for this is that the Te-DEGs were not specific; 37 of them were similarly regulated in the Tu samples (Figure 2C), albeit induction of these genes was usually much higher in Tu than in Te samples (Table S4). In leaves dual infested with *T. urticae* and *T. evansi*, the number of up-regulated DEGs was over 18% lower than in leaves solely infested with *T. urticae*. Furthermore, for numerous up-regulated genes in the Tu samples, the magnitude of induction was considerably lower in Tu+Te samples (Figure 2D). Both findings point to the suppression of *T. urticae*-induced plant responses by *T. evansi*. Indeed, various defense responses were antagonized by *T. evansi* at the transcriptome level, including genes coding for JA-responsive antinutritive proteins, SA-regulated PR proteins and enzymes associated with secondary metabolism (Figure 3, Figure 4, Figure 5 and Figure 6; Table 2). In this respect, two things are worthwhile to point out: Firstly, there was very little overlap in the JA defense genes that were suppressed by *A. lycopersici* versus by *T. evansi* (Figure 3). Secondly, the suppression of gene expression by *T. evansi* in dual-infested plants was evident even though the number of mites on leaflets of these plants was double that of the leaflets solely infested with *T. urticae* (thus excluding density-mediated effects). Given that (a) plant responses to mites, especially to defense-inducing ones, may vary over time [33,45] and (b) *T. urticae* is not immediately—or perhaps not always—facilitated by *T. evansi* on shared leaves [41,45], it would be interesting to analyze plant responses to dual attacks at early time points to find out how rapidly *T. urticae*-induced defenses are suppressed. Related to this, as *T. evansi*’s suppression trait appears to be plastic [41], it would be fascinating to explore the extent to which this plasticity is utilized to mitigate the facilitation of competing herbivores, such as *T. urticae* [50]. Finally, our most puzzling finding was that *T. evansi* not only suppressed *T. urticae*-induced genes, but also counteracted the *T. urticae*-triggered down-regulation of tomato genes (Figure 2D, Figure 4, Figure 5, Figure 6 and Figure 7; Table S9). Thus, *T. evansi* made dual-infested plants phenotypically more similar to non-infested plants via the transcriptome-wide dampening of *T. urticae*-triggered host responses. How exactly *T. evansi* achieves this is unknown [50]. We hypothesize that it secretes effector molecules that take control over central regulators of cellular homeostasis [50]. It is also conceivable that its effectors interfere with RNA polymerase activity [130] or silence host gene transcription epigenetically [131,132]. The ongoing identification and characterization of *in planta* targets of mite effectors [40,133,134] will undoubtedly allow us to better understand how the distinct transcriptomic profiles of suppressor mite-infested tomato plants come about. Once similar data has been obtained from other plant-herbivore/pathogen systems in which indirect plant-mediated interactions have been shown to modulate community ecology [14,15,16,17,19,20,21,22,24,25], it may be possible to identify common denominators as well as to assess if similar ecological problems (e.g., facilitating competitors) have been solved differently by organisms.

## 4. Material and Methods

### 4.1. Plants

Tomato (*S*. *lycopersicum* cv. Castlemart) and bean (*P*. *vulgaris* cv. Speedy) plants were germinated and grown in a greenhouse (25/18 °C day/night temperature, 16 light (L)/8 dark (D) photoperiod, 50–60% relative humidity (RH)). Experiments involving plants were carried out in a climate room (25 °C, 16 L/8 D photoperiod, 60% RH, 300 µmol m^−2^·s^−1^), to which plants were transferred seven days in advance.

### 4.2. Mites

We used spider mites (Acari: Tetranychidae) from the *T. urticae* Santpoort-2 and *T. evansi* Viçosa-1 strains [33]. When feeding from tomato, *T. urticae* Santpoort-2 mites induce JA and SA-regulated defenses, to which they are also susceptible [33,38,40,41], while *T. evansi* Viçosa-1 mites have been described to suppress these defenses [30,33,41]. Spider mites were reared on detached bean (for *T. urticae*) or tomato (for *T. evansi*) leaves in a climate room (25 °C, 16L/8D photoperiod, 60% RH, 300 µmol m^−2^·s^−1^). The tomato russet mites (*A. lycopersici*; Acari: Eriophyidae) we used, have been characterized before as suppressors of JA-regulated defenses and inducers of SA-regulated defenses, whilst being susceptible only to JA defenses [23]. Russet mites were reared on intact 21–35-day-old tomato plants in a climate room (27/25 °C day/night temperature, 16 L/8 D photoperiod, 60% RH, 300 µmol m^−2^·s^−1^). Both Tetranychid and Eriophyid mites pierce plant cells with their stylet-shaped mouthparts, inject pierced cells with saliva and then suck up their contents [126,135,136].

### 4.3. Tomato Infestation and Sampling

In order to measure phytohormone concentrations and plant defense gene expression upon the various mite infestations, mites were transferred onto 21-day-old, intact tomato plants and infested leaflets were harvested seven days later, following previously described procedures [23,33], with minor modifications. In short, plants were infested either with one species of mite (*T. urticae*, *T. evansi*, or *A. lycopersici*), referred to as “single infestations”, or simultaneously with a combination of two species (*T. urticae* plus *T. evansi*, or *T. urticae* plus *A. lycopersici*), referred to as “dual infestations”.

Spider mite infestations were performed by individually transferring adult females, randomly collected from the rearing colony, with a fine brush onto each of three leaflets per plant. For single infestations, each leaflet received 15 spider mites so that in total each plant was infested with 45 spider mites. Russet mite infestations were performed by transferring mites on small pieces (ca. 0.5 cm²) of leaflet, cut out from well-infested rearing-plants, onto each of three leaflets per plant. These leaflet pieces were cut out while using a stereo microscope (Leica MZ6; Leica Microsystems, Wetzlar, Germany) to ensure each of them contained ca. 250 mobile stages of russet mites. For single infestations, each plant was thus infested with ca. 750 russet mites. For dual infestations, each of three leaflets received either 15 *T. urticae* + *15 T. evansi* (in total 90 spider mites per plant), or 15 *T. urticae* + ca. 250 *A. lycopersici* (in total 45 spider mites and ca. 750 russet mites per plant). A lanolin (Sigma-Aldrich, St. Louis, MO, USA) barrier was made around the petiolule to prevent the mites from escaping. Leaflets from non-infested control plants also got a lanolin barrier.

Seven days after the infestations, infested leaflets and corresponding leaflets of non-infested control plants were excised, flash-frozen in liquid nitrogen, and stored at −80 °C until we extracted their phytohormones and isolated the RNA. Mites were not counted, nor removed, prior to the harvest of leaf material. Likewise, we did not quantify mite-inflicted feeding damage, because russet mite feeding activity cannot be quantified yet. The three leaflets obtained from the same plant were pooled to form one biological replicate. This experiment was replicated four times in consecutive weeks, each time using six plants per treatment, resulting in a total of 144 samples (6 treatments × 6 plants × 4 experimental replicates).

The mite densities and sampling moment used in this study were carefully selected based on previous studies [23,33,37,38] to maximize the opportunity to capture plant defense suppression by mites. That is, although the magnitude of defense responses induced by *T. urticae* increases as the infestation progresses, tomato leaflets infested with 15 of these mites usually enter senescence after eight to nine days and die shortly after [33]. Leaflets infested with 15 *T. evansi* do not senesce prematurely [33] and stay green (except for the emptied cells) and turgid for at least 14 days. Similarly, leaflets infested with 250 *A. lycopersici* do show clear signs of infestation (i.e., “silvering”) after seven days, but it takes roughly another week before they senesce [23]. The *T. urticae*-induced senescence was not dramatically accelerated in dual-infested leaves, therefore the sampling moment was set at seven days after introduction of the mites, i.e., when *T. urticae*-induced defense responses peak. Russet mite densities were higher than those of spider mites, because russet mites are much smaller and their per capita consumption rate is thus smaller as well [135,136]. Yet, we aimed to normalize mite densities on the basis of their estimated egg biomass production (since food is mostly converted into eggs). Lastly, we analyzed our data in a conservative manner by avoiding quantitative comparisons that could be explained simply by differences in mite densities.

### 4.4. Tomato Phytohormone Isolation and Analysis

Phytohormones were extracted from ca. 250 mg ground, homogenized leaf tissue per sample and then quantified by means of liquid chromatography-tandem mass spectrometry (LC-MS/MS) following previously described procedures [33]. Phytohormone data was analyzed with PASW Statistics 18 software (SPSS Ltd., Hong Kong, China). First, the combined data (all hormones) was used for a multivariate analysis of variance (MANOVA) with the “Pillai’s Trace” function. Next, data from individual hormones was analyzed with a generalized linear model (GLM), using a “γ” probability distribution, “log” link function, “treatment” as fixed factor and “biological replicate” nested within “treatment” in the model. When significant differences were found with the GLM, means of each group were compared using Fisher’s Least Significant Difference (LSD) post-hoc test.

### 4.5. Tomato RNA Isolation

The same leaf tissue samples that were used for phytohormone isolation were also used for RNA isolation. Total RNA was isolated from ca. 50–100 mg ground, homogenized leaf tissue per sample using the hot phenol method [137]. RNA integrity was checked by agarose-gel electrophoresis and a NanoDrop spectrophotometer (ND-1000; Thermo Fisher Scientific, Waltham, MA, USA) was subsequently used to assess RNA quantity and purity.

### 4.6. Microarray Hybridizations

For the microarray hybridizations, equal amounts of RNA, isolated from each of the six plants per treatment, were pooled, resulting in a total of 24 samples (6 treatments × 4 experimental replicates). RNA was hybridized on custom 12 × 135K microarrays (Roche NimbleGen, Basel, Switzerland), using one array per sample (pooled treatment). The probe sequences (60-mer oligo’s) were identical to those from the tomato Agilent 4 × 44K array (G2519F-022270; Agilent Technologies, Santa Clara, CA, USA) and additionally included ca. 600 custom sequences. Each probe was represented by three spots on each array. Individual samples were Cy3-labeled and these were normalized across arrays via a common reference design using a Cy5-labeled pool of all the samples on each array. Preparation, labeling, purification, hybridization and scanning was carried out by the MicroArray Department (MAD) of the University of Amsterdam (Amsterdam, the Netherlands) according to the manufacturer’s specifications (Roche NimbleGen).

### 4.7. Microarray Analysis

Cyanine signal intensity data were Log_2_-transformed and normalized (Loess and Aquantile). Prior to the final differential gene expression analysis, the 44,195 probe sequences were remapped to the ITAG3.2 gene annotation (dated 15 June 2017) of the tomato genome [138] using “Bowtie2–2.2.6” with default parameters [139]. The 32,182 probes (72.8% of the total probe number) that mapped to the annotated tomato genome were used in the downstream analyses. Using “limma” in the Bioconductor R environment [140], a linear model that treats the samples from non-infested control plants as a common reference was fitted to the processed data. Relative transcript levels and associated Benjamini-Hochberg false discovery rate-adjusted *p*-values were identified via empirical Bayes statistics. Significant differentially expressed genes (DEGs) were identified by applying a *p*-value and Log_2_ fold change (FC) cutoff of 0.05 and 0.585, respectively. The optimal number of clusters for the *k*-means clustering approach was assessed using the “gap statistics” [141] (method = “first max”; seed = “54321”). The *k*-means clustering was performed with centered Pearson’s correlation as distance metric. Based on the ITAG3.2 protein annotation [138], Biological Process (BP) Gene Ontology (GO) terms were ascribed to the 16,431 tomato genes that were analyzed within our gene-expression microarray approach using the Bioconductor packages “GO.db” [142] and “topGO” [143]. Two types of gene set enrichment analyses were conducted with the Bioconductor package “piano” [144]. First, the five transcriptomic responses associated with the different mite feeding regimes (Te vs. C, Tu+Te vs. C, Tu vs. C, Tu+Al vs. C, and Al vs. C) were investigated using the differential expression-associated statistics generated by the common-reference linear model in a distinct directional gene set analysis (PAGE). Second, GO-enrichment was also examined within the *k*-means-clustered gene sets using a one-tailed Fisher’s exact test. Tomato metabolic pathways were specifically investigated using the GOMapMan annotation [145], manually curated, and visualized by “gplots” and “ggplot2” [146]. Finally, to study the extent to which tomato genes are JA-responsive, we cross-referenced all genes on our gene-expression microarray with those that were significantly induced in the tomato JA-biosynthesis mutant *def-1*, 24 h after exogenous application of JA [39].

### 4.8. Real-Time Quantitative Reverse-Transcriptase Polymerase Chain Reaction (qPCR)

The RNA samples that were used for microarray hybridizations were also used for cDNA synthesis and subsequently for gene expression analysis by means of qPCR. We followed the same experimental setup as for the microarray hybridizations, meaning that equal amounts of RNA, isolated from each of the six plants per treatment, were pooled, resulting in a total of 24 samples (6 treatments × 4 experimental replicates). DNAse treatment, cDNA synthesis and qPCRs were performed as described previously [41]. qPCR data was analyzed with PASW Statistics 18 software (SPSS Ltd.) with a GLM, using a “γ” probability distribution and “log” link function. The model contained the following factors: “treatment” and “technical replicate” (i.e., two for each cDNA sample), the latter was nested within “biological replicate” [33]. When significant differences were found, means of each group were compared using Fisher’s LSD post-hoc test. Gene identifiers, primer sequences and references [41,61,88,107,113,114,147,148] are listed in Table S10.

## Figures and Tables

**Table 1:** List of candidate tomato (*Solanum lycopersicum*) genes whose expression may be suppressed by *Aculops lycopersici*, i.e., relative to full induction by *Tetranychus urticae*. Shown are Log_2_ fold change (Log_2_FC) values of tomato genes upon infestation with either a single mite species (*T. urticae* (Tu), *T. evansi* (Te), or *A. lycopersici* (Al)) or two species simultaneously (*T. urticae* plus *T. evansi* (Tu+Te), or *T. urticae* plus *A. lycopersici* (Tu+Al)) as compared to non-infested controls, along with their corresponding Benjamini and Hochberg false discovery rate adjusted *p* values (BH-adj *p*). Information in the column with header “JA” indicates whether expression of the respective gene was found to be significantly induced in the tomato JA-biosynthesis mutant *def-1*, 24 h after exogenous application of JA (for details see [39]). Candidate genes were selected based on two criteria: (1) expression is up-regulated (BH-adj *p* ≤ 0.05) in the Tu sample, while; (2) in the Tu+Al sample such up-regulation is considerably lower (Log_2_FC in Tu (shaded blue) − Log_2_FC in Tu+Al (shaded lilac) > 0.5) or absent. Selected genes were ranked according to their relative expression level in the Tu sample. Grey letters indicate a non-significant (BH-adjusted *p* > 0.05) change in gene expression.

				Te			Tu+Te			Tu			Tu+Al			Al	
#	Locus Identifier	Annotation (ITAG 3.2)	JA	Log_2_FC	BH-adj *p*		Log_2_FC	BH-adj *p*		Log_2_FC	BH-adj *p*		Log_2_FC	BH-adj *p*		Log_2_FC	BH-adj *p*
1	Solyc03g098760	Kunitz-type trypsin inhibitor	YES	2.85	<0.0001		4.64	<0.0001		4.70	<0.0001		3.48	<0.0001		2.16	<0.0001
2	Solyc03g098720	Kunitz-type trypsin inhibitor	YES	2.04	0.0104		3.70	<0.0001		3.69	<0.0001		1.59	0.0025		1.13	0.0527
3	Solyc04g074770	Unknown protein	YES	1.64	<0.0001		3.25	<0.0001		3.26	<0.0001		2.54	<0.0001		1.22	0.0001
4	Solyc03g020060	Proteinase inhibitor IId		0.91	0.6783		3.02	<0.0001		3.17	<0.0001		1.75	<0.0001		0.62	0.2031
5	Solyc10g078360	Short-chain dehydrogenase/reductase	YES	1.34	<0.0001		2.40	<0.0001		2.34	<0.0001		1.60	<0.0001		1.03	<0.0001
6	Solyc01g095960	Diacylglycerol O-acyltransferase	YES	0.23	0.9994		1.90	<0.0001		2.21	<0.0001		1.16	<0.0001		0.45	0.0687
7	Solyc03g098790	Jasmonate-inducible protein 21	YES	−0.26	0.9994		2.36	0.0001		2.18	0.0003		0.05	0.9582		−0.71	0.3061
8	Solyc12g010020	Leucine aminopeptidase A1	YES	1.58	0.0316		2.63	<0.0001		2.17	<0.0001		1.35	0.0024		0.70	0.1912
9	Solyc09g084480	Proteinase inhibitor I	YES	0.46	0.9994		2.23	<0.0001		2.10	<0.0001		0.50	0.2820		−0.12	0.8912
10	Solyc00g187050	Leucine aminopeptidase 2	YES	1.40	0.0595		2.47	<0.0001		2.06	<0.0001		1.15	0.0065		0.52	0.3414
11	Solyc09g089530	Proteinase inhibitor I	YES	−0.12	0.9994		1.79	0.0019		1.78	0.0013		−0.19	0.8004		−0.63	0.3401
12	Solyc09g084470	Proteinase inhibitor I	YES	−0.12	0.9994		1.73	0.0013		1.72	0.0009		0.22	0.7452		−0.55	0.3860
13	Solyc01g006400	Cysteine-rich extensin-like protein	YES	0.39	0.9994		1.76	<0.0001		1.65	<0.0001		0.52	0.1883		0.01	0.9868
14	Solyc01g091170	Arginase 2	YES	0.53	0.9994		1.60	<0.0001		1.53	<0.0001		0.72	0.0298		0.69	0.0563
15	Solyc10g084320	Subtilase	YES	0.48	0.9994		1.78	<0.0001		1.50	0.0002		0.90	0.0146		0.40	0.4223
16	Solyc09g089505	Proteinase inhibitor I		0.03	0.9994		1.48	0.0009		1.49	0.0005		0.11	0.8607		0.02	0.9817
17	Solyc12g010025	Leucine aminopeptidase		1.18	0.7638		1.73	0.0055		1.48	0.0142		0.67	0.2778		0.42	0.6154
18	Solyc09g089500	Proteinase inhibitor I	YES	0.15	0.9994		1.69	0.0010		1.40	0.0049		0.18	0.7905		−0.28	0.7205
19	Solyc02g071700	GDSL esterase/lipase	YES	0.51	0.9994		1.50	0.0061		1.32	0.0125		0.54	0.3295		−0.17	0.8606
20	Solyc04g018110	Calmodulin-like protein		0.31	0.9994		1.14	0.0420		1.31	0.0121		0.52	0.3539		1.05	0.0439
21	Solyc01g087840	Subtilase		0.18	0.9994		1.33	0.0042		1.30	0.0035		0.76	0.0707		0.41	0.4702
22	Solyc01g105650	2-Oxoglutarate and Fe(II)-dependent oxygenase		0.32	0.9994		1.12	<0.0001		1.25	<0.0001		0.71	0.0001		0.34	0.0970
23	Solyc06g083900	R2R3 MYB transcription factor 13	YES	0.55	0.9994		1.54	<0.0001		1.18	0.0010		0.51	0.1473		0.07	0.9186
24	Solyc01g006390	Cysteine-rich extensin-like protein	YES	0.40	0.9994		1.36	<0.0001		1.16	<0.0001		0.44	0.0801		0.01	0.9806
25	Solyc09g084490	Proteinase inhibitor I	YES	0.25	0.9994		1.01	0.0114		1.13	0.0027		0.23	0.6315		−0.10	0.8940
26	Solyc08g076980	Acetylornithine deacetylase	YES	0.38	0.9994		0.87	0.0323		0.96	0.0125		0.15	0.7688		−0.01	0.9929
27	Solyc01g006300	Peroxidase CEVI1		0.32	0.9994		0.87	0.0014		0.88	0.0008		0.33	0.2103		−0.07	0.8844
28	Solyc08g076970	Acetylornithine deacetylase	YES	0.37	0.9994		0.94	0.0210		0.83	0.0348		0.05	0.9272		0.02	0.9827
29	Solyc08g074630	Polyphenol oxidase F	YES	0.19	0.9994		1.00	0.0079		0.78	0.0357		0.10	0.8418		−0.09	0.8950
30	Solyc07g007250	Metallocarboxypeptidase inhibitor	YES	0.22	0.9994		0.82	0.0110		0.77	0.0134		−0.10	0.8219		−0.25	0.5489

**Table 2:** List of candidate tomato (*Solanum lycopersicum*) genes whose expression may be suppressed by *Tetranychus evansi*, i.e., relative to full induction by *T. urticae*. Shown are Log_2_ fold change (Log_2_FC) values of tomato genes upon infestation with either a single mite species (*T. urticae* (Tu), *T. evansi* (Te), or *Aculops lycopersici* (Al)) or two species simultaneously (*T. urticae* plus *T. evansi* (Tu+Te), or *T. urticae* plus *A. lycopersici* (Tu+Al)) as compared to non-infested controls, along with their corresponding Benjamini and Hochberg false discovery rate adjusted *p* values (BH-adj *p*). Information in the column with header “JA” indicates whether expression of the respective gene was found to be significantly induced in the tomato JA-biosynthesis mutant *def-1*, 24 h after exogenous application of JA (for details see [39]). Candidate genes were selected based on two criteria: (1) expression is up-regulated (BH-adj *p* ≤ 0.05) in the Tu sample, while; (2) in the Tu+Te sample such up-regulation is considerably lower (Log_2_FC in Tu (shaded blue) − Log_2_FC in Tu+Te (shaded yellow) > 0.5) or absent. Selected genes were ranked according to their relative expression level in the Tu sample. Grey letters indicate a non-significant (BH-adjusted *p* > 0.05) change in gene expression.

				Te			Tu+Te			Tu			Tu+Al			Al	
#	Locus Identifier	Annotation (ITAG 3.2)	JA	Log_2_FC	BH-adj *p*		Log_2_FC	BH-adj *p*		Log_2_FC	BH-adj *p*		Log_2_FC	BH-adj *p*		Log_2_FC	BH-adj *p*
1	Solyc10g083690	Cytochrome P450		0.54	0.9994		3.49	<0.0001		4.29	<0.0001		5.30	<0.0001		3.29	<0.0001
2	Solyc01g080570	Inosine/uridine-preferring nucleoside hydrolase		0.72	0.9994		3.61	<0.0001		4.22	<0.0001		4.65	<0.0001		3.29	<0.0001
3	Solyc04g064880	Pathogenesis-related protein		0.17	0.9994		3.28	<0.0001		3.92	<0.0001		4.90	<0.0001		2.58	<0.0001
4	Solyc01g060020	β-1,3-glucanase		0.43	0.9994		2.90	<0.0001		3.53	<0.0001		4.13	<0.0001		3.26	<0.0001
5	Solyc05g050350	Cyclic nucleotide-gated channel		0.97	0.0558		2.89	<0.0001		3.42	<0.0001		3.98	<0.0001		3.23	<0.0001
6	Solyc12g049030	Fatty acid desaturase		0.33	0.9994		2.75	0.0006		3.36	<0.0001		3.11	<0.0001		2.98	0.0001
7	Solyc01g059965	β-1,3-glucanase		0.34	0.9994		2.82	<0.0001		3.33	<0.0001		3.81	<0.0001		2.96	<0.0001
8	Solyc03g044830	Transducin/WD40 repeat-like protein		0.28	0.9994		2.64	<0.0001		3.23	<0.0001		3.79	<0.0001		2.57	<0.0001
9	Solyc10g078230	Cytochrome P450		−0.01	0.9994		2.66	<0.0001		3.17	<0.0001		4.32	<0.0001		3.16	<0.0001
10	Solyc11g007980	Cytochrome P450		0.13	0.9994		2.28	<0.0001		2.99	<0.0001		3.68	<0.0001		2.32	<0.0001
11	Solyc12g045020	Cytochrome P450		−0.09	0.9994		2.23	<0.0001		2.90	<0.0001		3.41	<0.0001		2.17	<0.0001
12	Solyc03g098740	Kunitz-type trypsin inhibitor		0.04	0.9994		2.27	0.0007		2.90	<0.0001		4.47	<0.0001		2.84	<0.0001
13	Solyc06g061215	Proteinase inhibitor II		0.02	0.9994		2.08	0.0002		2.67	<0.0001		3.43	<0.0001		1.43	0.0077
14	Solyc08g066880	5’-methylthioadenosine/S-adenosyl-homocysteine nucleosidase, putative	YES	−0.11	0.9994		1.87	0.0146		2.66	0.0002		3.45	<0.0001		0.93	0.2547
15	Solyc10g083700	Cytochrome P450		0.30	0.9994		1.83	0.0022		2.58	<0.0001		3.72	<0.0001		2.03	0.0003
16	Solyc10g083290	Extracellular invertase LIN6		0.36	0.9994		1.93	<0.0001		2.55	<0.0001		3.62	<0.0001		3.29	<0.0001
17	Solyc05g008220	Unknown protein		0.19	0.9994		2.00	<0.0001		2.55	<0.0001		3.58	<0.0001		2.18	<0.0001
18	Solyc02g093180	N-hydroxycinnamoyl/benzoyl-transferase	YES	0.67	0.9994		1.74	0.0041		2.37	0.0001		3.08	<0.0001		2.12	0.0002
19	Solyc03g020050	Proteinase inhibitor IIc	YES	0.13	0.9994		1.51	0.0010		2.20	<0.0001		2.15	<0.0001		0.54	0.2953
20	Solyc08g067610	ABC transporter		−0.13	0.9994		1.60	0.0004		2.18	<0.0001		2.72	<0.0001		1.34	0.0016
21	Solyc07g005100	Chitinase		0.26	0.9994		1.59	0.0001		2.11	<0.0001		3.05	<0.0001		2.34	<0.0001
22	Solyc06g066590	Unknown protein		0.05	0.9994		1.49	0.0001		2.04	<0.0001		2.83	<0.0001		1.89	<0.0001
23	Solyc03g098100	NAD(P)H-dependent oxidoreductase		0.38	0.9994		0.73	0.2857		1.53	0.0051		2.53	<0.0001		1.51	0.0043
24	Solyc04g016470	β-1,3-glucanase		−0.11	0.9994		0.71	0.1603		1.38	0.0014		1.56	0.0001		0.96	0.0269
25	Solyc11g007390	Glycosyltransferase		−0.04	0.9994		0.72	0.0791		1.38	0.0002		1.98	<0.0001		0.98	0.0061
26	Solyc03g020010	Kunitz-type trypsin inhibitor		−0.17	0.9994		0.82	0.1136		1.34	0.0031		2.71	<0.0001		1.60	0.0003
27	Solyc07g006500	Trehalose-6-phosphate synthase		−0.16	0.9994		0.76	0.1422		1.34	0.0027		2.11	<0.0001		0.96	0.0310
28	Solyc11g044910	β-xylosidase	YES	−0.29	0.9994		0.54	0.3539		1.29	0.0040		1.95	<0.0001		0.83	0.0698
29	Solyc08g078650	Glycosyltransferase		−0.02	0.9994		0.67	0.0368		1.19	0.0001		1.73	<0.0001		0.89	0.0019
30	Solyc07g045000	Unknown protein		−0.11	0.9994		0.49	0.1225		1.05	0.0001		1.73	<0.0001		0.72	0.0072
31	Solyc09g082230	Ribosomal-protein-alanine N-acetyl-transferase		0.22	0.9994		0.05	0.9354		0.61	0.0273		0.62	0.0128		0.64	0.0166

## Data Availability

Tomato transcriptomic data has been uploaded to the Gene Expression Omnibus repository (accession number GSE116827). Tomato phytohormone and qPCR data has been uploaded to FigShare (10.6084/m9.figshare.7195097). All raw data is publicly available.

## Supplementary Materials

Supplementary materials can be found at http://www.mdpi.com/1422-0067/19/10/3265/s1.
