# Supplementary material for: Distinct Signatures of Host Defense Suppression by Plant-Feeding Mites
Source: Int J Mol Sci. 2018 Oct 20;19(10):3265. doi: 10.3390/ijms19103265 (PMC6214137; doi:10.3390/ijms19103265)
Supplement: Supplementary file 1 [file ijms-19-03265-s001.pdf]

International Journal of *Molecular Sciences*

MDPI

*Supplementary Materials*

# Distinct Signatures of Host Defense Suppression by Plant-Feeding Mites

**Figure S1.** Relative transcript abundances of defense-associated tomato (*Solanum lycopersicum*) genes in leaflets after seven days of infestation with herbivorous mites. Tomato leaves were infested with either a single mite species (*Tetranychus urticae* [Tu], *Tetranychus evansi* [Te], or *Aculops lycopersici* [Al]) or two species simultaneously (*T. urticae* plus *T. evansi* [Tu+Te], or *T. urticae* plus *A. lycopersici* [Tu+Al]). Non-infested plants served as controls [C]. The figure shows the average (+ SEM) normalized transcript abundances of: (**A**) *Jasmonate-inducible protein 21* (*JIP-21*); (**B**) *Proteinase inhibitor IIc* (*PI-IIc*); (**C**) *Kunitz-type trypsin inhibitor* (*KTI_20*); (**D**) *Kunitz-type trypsin inhibitor* (*KTI_40*); (**E**) *Leucine aminopeptidase A1* (*LapA1*); (**F**) *Cytochrome P450* (*CYP450*); (**G**) *Serine proteinase inhibitor* (*Ser PI*); (**H**) *Isochorismate synthase* (*ICS*); (**I**) *β-1-3-glucanase* (*β-1-3-gluc*), and; (**J**) *Pathogenesis-related protein* (*PR-P*), as determined by qPCR. Transcript abundances were normalized to *Actin* and then scaled to the overall lowest average value per gene panel. Different letters above the bars indicate significant differences at a level of $P \leq 0.05$, after applying a generalized linear model followed by Fisher's Least Significant Difference test.

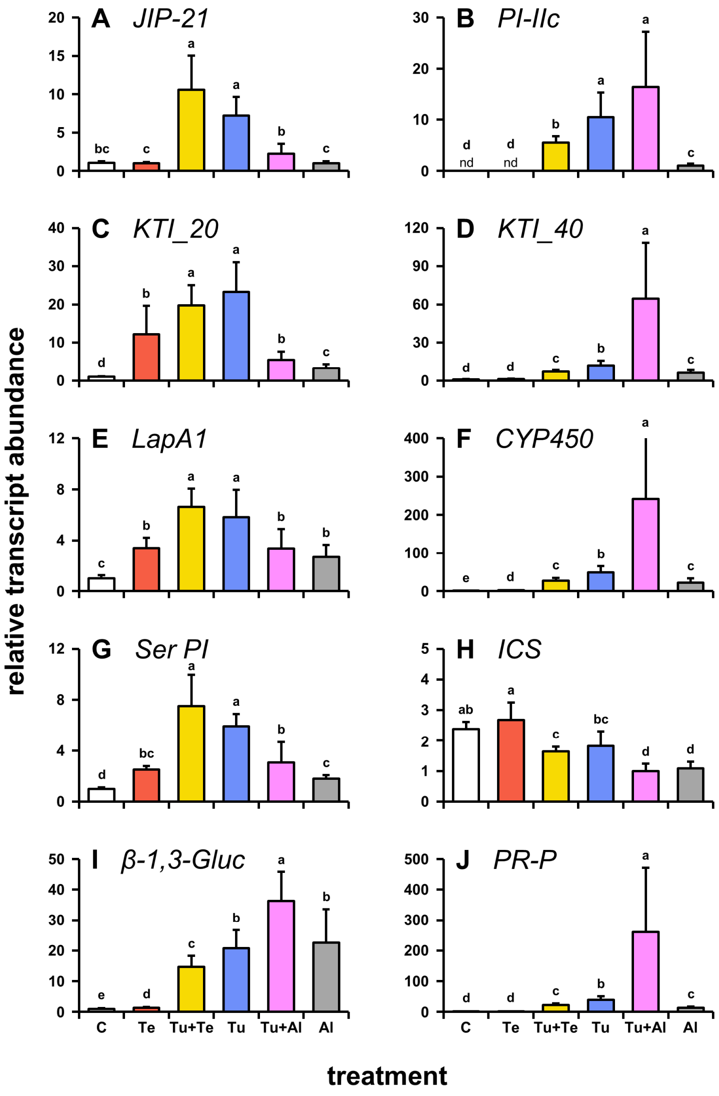

**Figure S2.** Relative transcript abundances of flavonoid and steroidal (glycol)alkaloid biosynthetic tomato (*Solanum lycopersicum*) genes in leaflets after seven days of infestation with herbivorous mites. Tomato leaves were infested with either a single mite species (*Tetranychus urticae* [Tu], *Tetranychus evansi* [Te], or *Aculops lycopersici* [Al]) or two species simultaneously (*T. urticae* plus *T. evansi* [Tu+Te], or *T. urticae* plus *A. lycopersici* [Tu+Al]). Non-infested plants served as controls [C]. The figure shows the average (+ SEM) normalized transcript abundances of: (**A**) *Chalcone synthase 1* (*CHS1*); (**B**) *Chalcone synthase 2* (*CHS2*); (**C**) *Glycoalkaloid metabolism 1* (*GAME1*), and; (**D**) *Glycoalkaloid metabolism 9 / Jasmonate-responsive ethylene response factor 4* (*GAME9/JRE4*), as determined by qPCR. Transcript abundances were normalized to *Actin* and then scaled to the overall lowest average value per gene panel. Different letters above the bars indicate significant differences at a level of $P \leq 0.05$, after applying a generalized linear model followed by Fisher's Least Significant Difference test.

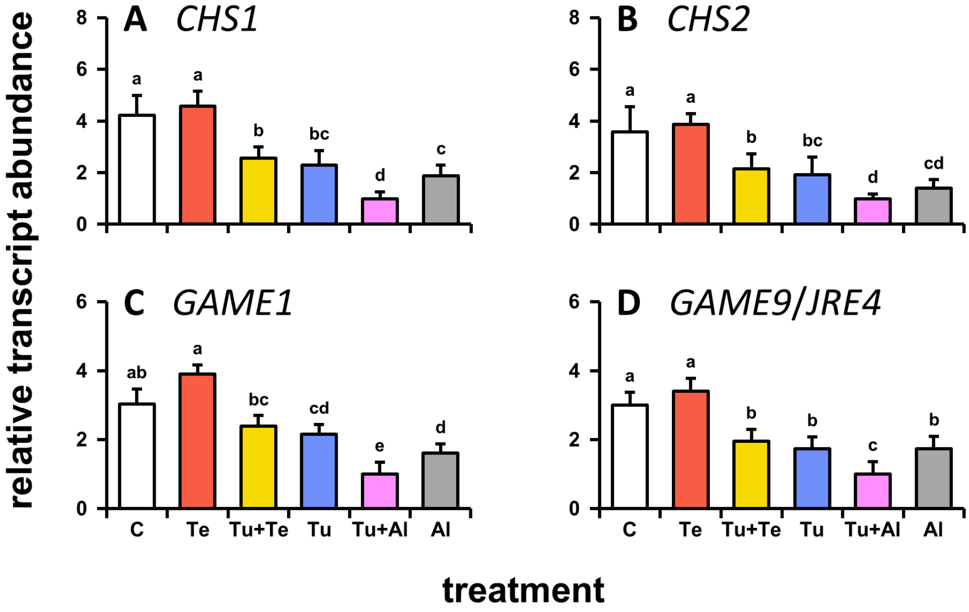


**Figure S3.** Gene-expression heat map depicting the relative transcript levels of tomato (*Solanum lycopersicum*) genes that encode proteins with a (predicted) function in phytohormone biosynthesis/signaling and that were differentially expressed in leaves after seven days of infestation with herbivorous mites. Tomato leaves were infested with either a single mite species (*Tetranychus urticae* [Tu], *Tetranychus evansi* [Te], or *Aculops lycopersici* [Al]) or two species simultaneously (*T. urticae* plus *T. evansi* [Tu+Te], or *T. urticae* plus *A. lycopersici* [Tu+Al]). Non-infested plants served as controls and were used as a common reference in the transcriptional comparisons. The figure shows heat maps for the following phytohormones: (**A**) abscisic acid, (**B**) auxin, (**C**) ethylene, (**D**) brassinosteroids, (**E**) gibberellins, and (**F**) cytokinins. Presented genes were differentially expressed (Benjamini and Hochberg false discovery rate adjusted $P \leq 0.05$; $Log_2$ fold change (FC) ≥ 0.585) in at least one of the mite-infestation treatments. The different (sub)sections of each pathway are specified on the left.

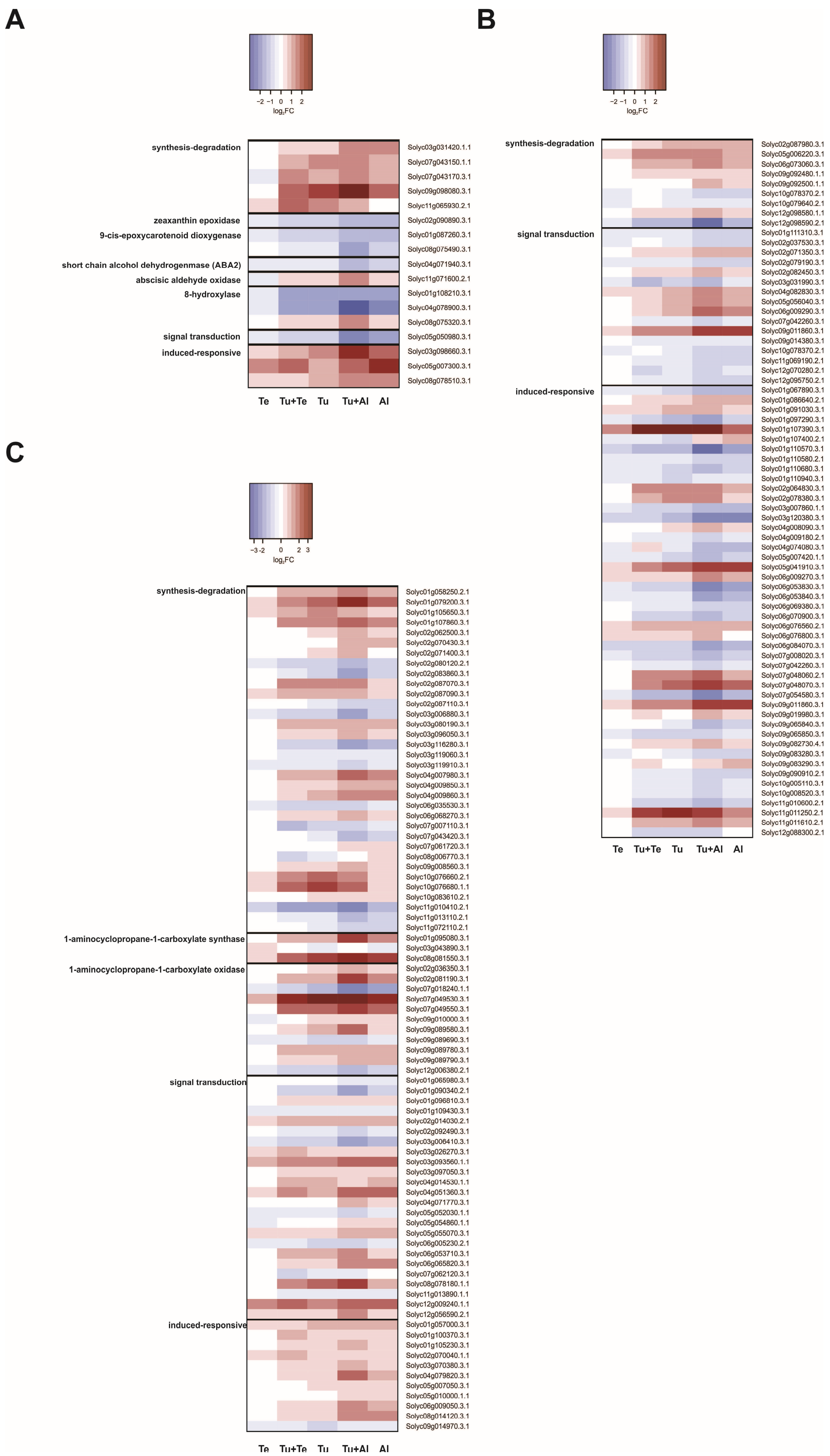

A
−2 −1 0 1 2
log2FC
synthesis-degradation
zeaxanthin epoxidase
9-cis-epoxycarotenoid dioxygenase
short chain alcohol dehydrogenmase (ABA2)
abscisic aldehyde oxidase
8-hydroxylase
signal transduction
induced-responsive
Solyc03g031420.1.1
Solyc07g043150.1.1
Solyc07g043170.3.1
Solyc09g098080.3.1
Solyc11g065930.2.1
Solyc02g090890.3.1
Solyc01g087260.3.1
Solyc08g075490.3.1
Solyc04g071940.3.1
Solyc11g071600.2.1
Solyc01g108210.3.1
Solyc04g078900.3.1
Solyc08g075320.3.1
Solyc05g050980.3.1
Solyc03g098660.3.1
Solyc05g007300.3.1
Solyc08g078510.3.1
Te Tu+Te Tu Tu+Al Al
B
−2 −1 0 1 2
log2FC
synthesis-degradation
signal transduction
induced-responsive
Solyc02g087980.3.1
Solyc05g006220.3.1
Solyc06g073060.3.1
Solyc09g092480.1.1
Solyc09g092500.1.1
Solyc10g078370.2.1
Solyc10g079640.2.1
Solyc12g098580.1.1
Solyc12g098590.2.1
Solyc01g111310.3.1
Solyc02g037530.3.1
Solyc02g071350.3.1
Solyc02g079190.3.1
Solyc02g082450.3.1
Solyc03g031990.3.1
Solyc04g082830.3.1
Solyc05g056040.3.1
Solyc06g009290.3.1
Solyc07g042260.3.1
Solyc09g011860.3.1
Solyc09g014380.3.1
Solyc10g078370.2.1
Solyc11g069190.2.1
Solyc12g070280.2.1
Solyc12g095750.2.1
Solyc01g067890.3.1
Solyc01g086640.2.1
Solyc01g091030.3.1
Solyc01g097290.3.1
Solyc01g107390.3.1
Solyc01g107400.2.1
Solyc01g110570.3.1
Solyc01g110580.2.1
Solyc01g110680.3.1
Solyc01g110940.3.1
Solyc02g064830.3.1
Solyc02g078380.3.1
Solyc03g007860.1.1
Solyc03g120380.3.1
Solyc04g008090.3.1
Solyc04g009180.2.1
Solyc04g074080.3.1
Solyc05g007420.1.1
Solyc05g041910.3.1
Solyc06g009270.3.1
Solyc06g053830.3.1
Solyc06g053840.3.1
Solyc06g069380.3.1
Solyc06g070900.3.1
Solyc06g076560.2.1
Solyc06g076800.3.1
Solyc06g084070.3.1
Solyc07g008020.3.1
Solyc07g042260.3.1
Solyc07g048060.2.1
Solyc07g048070.3.1
Solyc07g054580.3.1
Solyc09g011860.3.1
Solyc09g019980.3.1
Solyc09g065840.3.1
Solyc09g065850.3.1
Solyc09g082730.4.1
Solyc09g083280.3.1
Solyc09g083290.3.1
Solyc09g090910.2.1
Solyc10g005110.3.1
Solyc10g008520.3.1
Solyc11g010600.2.1
Solyc11g011250.2.1
Solyc11g011610.2.1
Solyc12g088300.2.1
Te Tu+Te Tu Tu+Al Al
C
−3 −2 0 2 3
log2FC
synthesis-degradation
1-aminocyclopropane-1-carboxylate synthase
1-aminocyclopropane-1-carboxylate oxidase
signal transduction
induced-responsive
Solyc01g058250.2.1
Solyc01g079200.3.1
Solyc01g105650.3.1
Solyc01g107860.3.1
Solyc02g062500.3.1
Solyc02g070430.3.1
Solyc02g071400.3.1
Solyc02g080120.2.1
Solyc02g083860.3.1
Solyc02g087070.3.1
Solyc02g087090.3.1
Solyc02g087110.3.1
Solyc03g006880.3.1
Solyc03g080190.3.1
Solyc03g096050.3.1
Solyc03g116280.3.1
Solyc03g119060.3.1
Solyc03g119910.3.1
Solyc04g007980.3.1
Solyc04g009850.3.1
Solyc04g009860.3.1
Solyc06g035530.3.1
Solyc06g068270.3.1
Solyc07g007110.3.1
Solyc07g043420.3.1
Solyc07g061720.3.1
Solyc08g006770.3.1
Solyc09g008560.3.1
Solyc10g076660.2.1
Solyc10g076680.1.1
Solyc10g083610.2.1
Solyc11g010410.2.1
Solyc11g013110.2.1
Solyc11g072110.2.1
Solyc01g095080.3.1
Solyc03g043890.3.1
Solyc08g081550.3.1
Solyc02g036350.3.1
Solyc02g081190.3.1
Solyc07g018240.1.1
Solyc07g049530.3.1
Solyc07g049550.3.1
Solyc09g010000.3.1
Solyc09g089580.3.1
Solyc09g089690.3.1
Solyc09g089780.3.1
Solyc09g089790.3.1
Solyc12g006380.2.1
Solyc01g065980.3.1
Solyc01g090340.2.1
Solyc01g096810.3.1
Solyc01g109430.3.1
Solyc02g014030.2.1
Solyc02g092490.3.1
Solyc03g006410.3.1
Solyc03g026270.3.1
Solyc03g093560.1.1
Solyc03g097050.3.1
Solyc04g014530.1.1
Solyc04g051360.3.1
Solyc04g071770.3.1
Solyc05g052030.1.1
Solyc05g054860.1.1
Solyc05g055070.3.1
Solyc06g005230.2.1
Solyc06g053710.3.1
Solyc06g065820.3.1
Solyc07g062120.3.1
Solyc08g078180.1.1
Solyc11g013890.1.1
Solyc12g009240.1.1
Solyc12g056590.2.1
Solyc01g057000.3.1
Solyc01g100370.3.1
Solyc01g105230.3.1
Solyc02g070040.1.1
Solyc03g070380.3.1
Solyc04g079820.3.1
Solyc05g007050.3.1
Solyc05g010000.1.1
Solyc06g009050.3.1
Solyc08g014120.3.1
Solyc09g014970.3.1
Te Tu+Te Tu Tu+Al Al

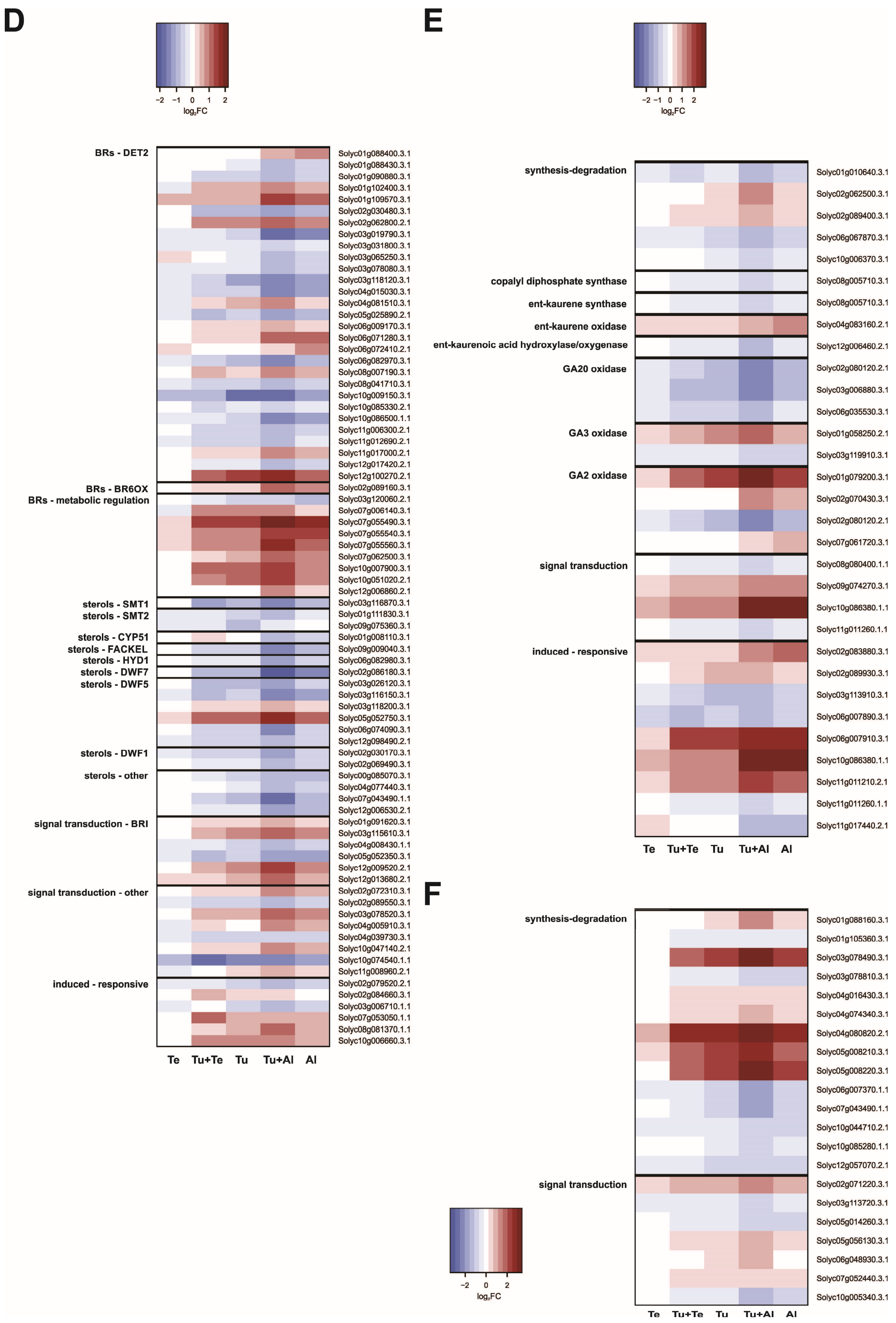
D
−2 −1 0 1 2
log2FC
BRs - DET2
BRs - BR6OX
BRs - metabolic regulation
sterols - SMT1
sterols - SMT2
sterols - CYP51
sterols - FACKEL
sterols - HYD1
sterols - DWF7
sterols - DWF5
sterols - DWF1
sterols - other
signal transduction - BRI
signal transduction - other
induced - responsive
Solyc01g088400.3.1
Solyc01g088430.3.1
Solyc01g090880.3.1
Solyc01g102400.3.1
Solyc01g109570.3.1
Solyc02g030480.3.1
Solyc02g062800.2.1
Solyc03g019790.3.1
Solyc03g031800.3.1
Solyc03g065250.3.1
Solyc03g078080.3.1
Solyc03g118120.3.1
Solyc04g015030.3.1
Solyc04g081510.3.1
Solyc05g025890.2.1
Solyc06g009170.3.1
Solyc06g071280.3.1
Solyc06g072410.2.1
Solyc06g082970.3.1
Solyc08g007190.3.1
Solyc08g041710.3.1
Solyc10g009150.3.1
Solyc10g085330.2.1
Solyc10g086500.1.1
Solyc11g006300.2.1
Solyc11g012690.2.1
Solyc11g017000.2.1
Solyc12g017420.2.1
Solyc12g100270.2.1
Solyc02g089160.3.1
Solyc03g120060.2.1
Solyc07g006140.3.1
Solyc07g055490.3.1
Solyc07g055540.3.1
Solyc07g055560.3.1
Solyc07g062500.3.1
Solyc10g007900.3.1
Solyc10g051020.2.1
Solyc12g006860.2.1
Solyc03g116870.3.1
Solyc01g111830.3.1
Solyc09g075360.3.1
Solyc01g008110.3.1
Solyc09g009040.3.1
Solyc06g082980.3.1
Solyc02g086180.3.1
Solyc03g026120.3.1
Solyc03g116150.3.1
Solyc03g118200.3.1
Solyc05g052750.3.1
Solyc06g074090.3.1
Solyc12g098490.2.1
Solyc02g030170.3.1
Solyc02g069490.3.1
Solyc00g085070.3.1
Solyc04g077440.3.1
Solyc07g043490.1.1
Solyc12g006530.2.1
Solyc01g091620.3.1
Solyc03g115610.3.1
Solyc04g008430.1.1
Solyc05g052350.3.1
Solyc12g009520.2.1
Solyc12g013680.2.1
Solyc02g072310.3.1
Solyc02g089550.3.1
Solyc03g078520.3.1
Solyc04g005910.3.1
Solyc04g039730.3.1
Solyc10g047140.2.1
Solyc10g074540.1.1
Solyc11g008960.2.1
Solyc02g079520.2.1
Solyc02g084660.3.1
Solyc03g006710.3.1
Solyc07g053050.1.1
Solyc08g081370.1.1
Solyc10g006660.3.1
Te Tu+Te Tu Tu+Al Al
E
−2 −1 0 1 2
log2FC
synthesis-degradation
copalyl diphosphate synthase
ent-kaurene synthase
ent-kaurene oxidase
ent-kaurenoic acid hydroxylase/oxygenase
GA20 oxidase
GA3 oxidase
GA2 oxidase
signal transduction
induced - responsive
Solyc01g010640.3.1
Solyc02g062500.3.1
Solyc02g089400.3.1
Solyc06g067870.3.1
Solyc10g006370.3.1
Solyc08g005710.3.1
Solyc08g005710.3.1
Solyc04g083160.2.1
Solyc12g006460.2.1
Solyc02g080120.2.1
Solyc03g006880.3.1
Solyc06g035530.3.1
Solyc01g058250.2.1
Solyc03g119910.3.1
Solyc01g079200.3.1
Solyc02g070430.3.1
Solyc02g080120.2.1
Solyc07g061720.3.1
Solyc08g080400.1.1
Solyc09g074270.3.1
Solyc10g086380.1.1
Solyc11g011260.1.1
Solyc02g083880.3.1
Solyc02g089930.3.1
Solyc03g113910.3.1
Solyc06g007890.3.1
Solyc06g007910.3.1
Solyc10g086380.1.1
Solyc11g011210.2.1
Solyc11g011260.1.1
Solyc11g017440.2.1
Te Tu+Te Tu Tu+Al Al
F
synthesis-degradation
signal transduction
Solyc01g088160.3.1
Solyc01g105360.3.1
Solyc03g078490.3.1
Solyc03g078810.3.1
Solyc04g016430.3.1
Solyc04g074340.3.1
Solyc04g080820.2.1
Solyc05g008210.3.1
Solyc05g008220.3.1
Solyc06g007370.1.1
Solyc07g043490.1.1
Solyc10g044710.2.1
Solyc10g085280.1.1
Solyc12g057070.2.1
Solyc02g071220.3.1
Solyc03g113720.3.1
Solyc05g014260.3.1
Solyc05g056130.3.1
Solyc06g048930.3.1
Solyc07g052440.3.1
Solyc10g005340.3.1
−2 0 2
log2FC
Te Tu+Te Tu Tu+Al Al

**Table S1.** Overview of microarray data, showing the transcriptomic changes in tomato (*Solanum lycopersicum*) leaves after seven days of infestation with either a single mite species (*Tetranychus urticae* [Tu], *T. evansi* [Te], or *Aculops lycopersici* [Al]) or two species simultaneously (*T. urticae* plus *T. evansi* [Tu+Te], or *T. urticae* plus *A. lycopersici* [Tu+Al]) as compared to non-infested controls. Differentially expressed genes (DEGs) were identified based on a Benjamini and Hochberg false discovery rate adjusted $P \leq 0.05$. ▲, up-regulated; ▼, down-regulated

| Treatment | All DEGs ▲ | All DEGs ▼ | All DEGs Total | DEGs at absolute fold change ≥ 1.5 ▲ | DEGs at absolute fold change ≥ 1.5 ▼ | DEGs at absolute fold change ≥ 1.5 Total |
|---|---|---|---|---|---|---|
| **Te** | 37 | 1 | 38 | 35 | 1 | 36 |
| **Tu+Te** | 1,049 | 983 | 2,032 | 795 | 530 | 1,325 |
| **Tu** | 1,281 | 1,179 | 2,460 | 925 | 631 | 1,556 |
| **Tu+Al** | 2,517 | 2,635 | 5,132 | 1,678 | 1,672 | 3,350 |
| **Al** | 1,676 | 1,524 | 3,200 | 1,159 | 874 | 2,033 |

**Table S2.** Overview of microarray data, showing the results from a gene set enrichment analysis (GSA) of biological process (BP) gene ontology (GO) annotations per mite-infestation treatment. We surveyed transcriptomic changes in tomato (*Solanum lycopersicum*) leaves after seven days of infestation with either a single mite species (*Tetranychus urticae* [Tu], *T. evansi* [Te], or *Aculops lycopersici* [Al]) or two species simultaneously (*T. urticae* plus *T. evansi* [Tu+Te], or *T. urticae* plus *A. lycopersici* [Tu+Al]) as compared to non-infested controls. Differentially expressed genes (DEGs) were identified based on a Benjamini and Hochberg (BH) false discovery rate adjusted $P \leq 0.05$ and an absolute fold change ≥ 1.5. The DEGs of each individual mite-infestation treatment were subsequently analyzed for enrichment of BP GO annotations using the PAGE algorithm (BH-adj $P \leq 0.05$). The Te treatment was excluded, because the number of Te-DEGs was insufficient for statistical analysis. (**A**) Results of the GSA for the up-regulated DEGs and (**B**) for the down-regulated DEGs per treatment. Values inside the columns annotated with 'DEGs' denote the number of up-regulated (▲) versus down-regulated (▼) DEGs associated with a particular GO term.

**(A) GSA for up-regulated DEGs of each treatment**

| | | Tu+Te | | Tu | | Tu+Al | | Al | |
|---|---|---|---|---|---|---|---|---|---|
| GO ID | GO term | DEGs ▲ / ▼ | BH-adj *P* | DEGs ▲ / ▼ | BH-adj *P* | DEGs ▲ / ▼ | BH-adj *P* | DEGs ▲ / ▼ | BH-adj *P* |
| GO:0006468 | protein phosphorylation | 64 / 19 | 0.0204 | 79 / 22 | 0.0003 | 145 / 54 | 0.0033 | 104 / 31 | 0.0046 |
| GO:0042744 | hydrogen peroxide catabolic process | 14 / 0 | 0.0200 | 16 / 2 | 0.0419 | 17 / 5 | 0.0044 | 13 / 3 | 0.0107 |
| GO:0048544 | recognition of pollen | 10 / 0 | 0.0259 | 11 / 0 | 0.0324 | 15 / 1 | 0.0033 | 10 / 0 | 0.0046 |
| GO:0006032 | chitin catabolic process | 6 / 0 | 0.0432 | 7 / 0 | 0.0419 | 8 / 0 | 0.0066 | 7 / 0 | 0.0359 |
| GO:0006629 | lipid metabolic process | 14 / 3 | 0.0200 | 16 / 5 | 0.0324 | 22 / 15 | 0.0451 | 19 / 8 | 0.0552 |
| GO:0019752 | carboxylic acid metabolic process | 5 / 1 | 0.0120 | 6 / 3 | 0.0756 | 6 / 4 | 0.0451 | 6 / 1 | 0.0264 |
| GO:0006950 | response to stress | 11 / 0 | 0.0277 | 12 / 0 | 0.0419 | 18 / 5 | 0.0264 | 14 / 3 | 0.0678 |
| GO:0006952 | defense response | 7 / 0 | 0.0259 | 6 / 1 | 0.0419 | 10 / 7 | 0.2324 | 6 / 2 | 0.0581 |
| GO:0009607 | response to biotic stimulus | 5 / 0 | 0.0259 | - | - | 5 / 5 | 0.2653 | 5 / 1 | 0.0376 |
| GO:0055114 | oxidation-reduction process | 96 / 41 | 0.0204 | 107 / 51 | 0.0324 | 161 / 155 | 0.3173 | 107 / 90 | 0.9431 |
| GO:0016998 | cell wall macromolecule catabolic process | - | - | 5 / 0 | 0.0419 | 5 / 0 | 0.0172 | 5 / 0 | 0.0552 |
| GO:0006811 | ion transport | - | - | 5 / 0 | 0.0419 | 5 / 1 | 0.0451 | 5 / 0 | 0.0552 |
| GO:0006457 | protein folding | 5 / 2 | 0.2463 | 5 / 0 | 0.0419 | 9 / 9 | 0.5074 | 7 / 1 | 0.0678 |
| GO:0006979 | response to oxidative stress | 18 / 3 | 0.0532 | 20 / 6 | 0.1419 | 23 / 14 | 0.0451 | 16 / 7 | 0.0678 |
| GO:0006869 | lipid transport | 6 / 0 | 0.0713 | 6 / 0 | 0.1419 | 8 / 2 | 0.0451 | 6 / 1 | 0.0678 |
| GO:0009664 | plant-type cell wall organization | - | - | - | - | 7 / 1 | 0.0451 | - | - |

**(B) GSA for down-regulated DEGs of each treatment**

| GO ID | GO term | Tu+Te DEGs ▲ / ▼ | Tu+Te BH-adj *P* | Tu DEGs ▲ / ▼ | Tu BH-adj *P* | Tu+Al DEGs ▲ / ▼ | Tu+Al BH-adj *P* | Al DEGs ▲ / ▼ | Al BH-adj *P* |
|---|---|---|---|---|---|---|---|---|---|
| GO:0015979 | photosynthesis | 0 / 12 | 0.0001 | 0 / 14 | 0.0001 | 0 / 38 | 0.0001 | 0 / 32 | 0.0001 |
| GO:0009765 | photosynthesis. light harvesting | - | - | 0 / 7 | 0.0021 | 0 / 17 | 0.0001 | 0 / 17 | 0.0001 |
| GO:0015995 | chlorophyll biosynthetic process | - | - | - | - | 0 / 6 | 0.0286 | 0 / 6 | 0.0313 |
| GO:0006096 | glycolytic process | - | - | - | - | 2 / 9 | 0.1529 | 0 / 6 | 0.0313 |
| GO:0008152 | metabolic process | 44 / 22 | 0.9997 | 48 / 32 | 0.9998 | 76 / 82 | 0.7245 | 47 / 54 | 0.0313 |

**Table S3.** List of the 25 most highly up-regulated genes in tomato (*Solanum lycopersicum*) leaves after seven days of infestation with *Tetranychus urticae*. Shown are $Log_2$ fold change ($Log_2FC$) values, representing relative expression levels of tomato genes upon infestation with either a single mite species (*T. urticae* [Tu], *T. evansi* [Te], or *Aculops lycopersici* [Al]) or two species simultaneously (*T. urticae* plus *T. evansi* [Tu+Te], or *T. urticae* plus *A. lycopersici* [Tu+Al]) as compared to non-infested controls, along with their corresponding Benjamini and Hochberg false discovery rate adjusted *P* values (BH-adj *P*). Information in the column with header 'JA' indicates whether expression of the respective gene was found to be significantly induced in the tomato JA-biosynthesis mutant *def-1*, 24 hours after exogenous application of JA (for details see [39]). The asterisk denotes that JA-inducibility of the respective gene was established by [75]. Genes were ranked according to their relative expression level in the Tu sample (shaded grey).

| | | | | Te | | Tu+Te | | Tu | | Tu+Al | | Al | |
|---|---|---|---|---|---|---|---|---|---|---|---|---|---|
| **#** | **Locus identifier** | **Annotation (ITAG 3.2)** | **JA** | **$Log_2FC$** | **BH-adj *P*** | **$Log_2FC$** | **BH-adj *P*** | **$Log_2FC$** | **BH-adj *P*** | **$Log_2FC$** | **BH-adj *P*** | **$Log_2FC$** | **BH-adj *P*** |
| 1 | Solyc03g098760 | Kunitz-type trypsin inhibitor | YES | 2.85 | < 0.0001 | 4.64 | < 0.0001 | 4.70 | < 0.0001 | 3.48 | < 0.0001 | 2.16 | < 0.0001 |
| 2 | Solyc10g005320 | Tryptophan synthase | YES | 1.81 | 0.0004 | 4.14 | < 0.0001 | 4.36 | < 0.0001 | 4.31 | < 0.0001 | 2.41 | < 0.0001 |
| 3 | Solyc10g083690 | Cytochrome P450 | | 0.54 | 0.9994 | 3.49 | < 0.0001 | 4.29 | < 0.0001 | 5.30 | < 0.0001 | 3.29 | < 0.0001 |
| 4 | Solyc01g080570 | Inosine/uridine-preferring nucleoside hydrolase | | 0.72 | 0.9994 | 3.61 | < 0.0001 | 4.22 | < 0.0001 | 4.65 | < 0.0001 | 3.29 | < 0.0001 |
| 5 | Solyc04g064880 | Pathogenesis-related protein | | 0.17 | 0.9994 | 3.28 | < 0.0001 | 3.92 | < 0.0001 | 4.90 | < 0.0001 | 2.58 | < 0.0001 |
| 6 | Solyc05g014590 | bHLH DNA-binding protein | | 2.19 | < 0.0001 | 3.77 | < 0.0001 | 3.89 | < 0.0001 | 4.49 | < 0.0001 | 3.78 | < 0.0001 |
| 7 | Solyc01g106620 | Pathogenesis-related protein 1 | | 0.51 | 0.9994 | 3.38 | < 0.0001 | 3.83 | < 0.0001 | 4.09 | < 0.0001 | 2.72 | < 0.0001 |
| 8 | Solyc01g105450 | ABC transporter | YES | 1.55 | 0.0014 | 3.32 | < 0.0001 | 3.79 | < 0.0001 | 3.38 | < 0.0001 | 2.36 | < 0.0001 |
| 9 | Solyc01g106630 | CBL-interacting protein kinase 23 | | 0.49 | 0.9994 | 3.35 | < 0.0001 | 3.79 | < 0.0001 | 4.14 | < 0.0001 | 2.46 | < 0.0001 |
| 10 | Solyc03g098720 | Kunitz-type trypsin inhibitor | YES | 2.04 | 0.0104 | 3.70 | < 0.0001 | 3.69 | < 0.0001 | 1.59 | 0.0025 | 1.13 | 0.0527 |
| 11 | Solyc09g091550 | Salicylic acid methyltransferase | YES* | 1.59 | < 0.0001 | 3.58 | < 0.0001 | 3.64 | < 0.0001 | 3.78 | < 0.0001 | 2.87 | < 0.0001 |
| 12 | Solyc08g074682 | Polyphenol oxidase precursor | | 1.35 | 0.0104 | 3.52 | < 0.0001 | 3.60 | < 0.0001 | 3.61 | < 0.0001 | 0.85 | 0.0240 |
| 13 | Solyc01g060020 | β-1,3-glucanase | | 0.43 | 0.9994 | 2.90 | < 0.0001 | 3.53 | < 0.0001 | 4.13 | < 0.0001 | 3.26 | < 0.0001 |
| 14 | Solyc08g067630 | Histone deacetylase-like protein | | 1.58 | 0.1823 | 3.31 | < 0.0001 | 3.52 | < 0.0001 | 4.37 | < 0.0001 | 3.39 | < 0.0001 |
| 15 | Solyc01g059990 | Serine/threonine protein phosphatase | | 0.34 | 0.9994 | 3.03 | < 0.0001 | 3.51 | < 0.0001 | 3.94 | < 0.0001 | 3.15 | < 0.0001 |
| 16 | Solyc01g059980 | β-1,3-glucanase | | 0.36 | 0.9994 | 3.03 | < 0.0001 | 3.45 | < 0.0001 | 3.96 | < 0.0001 | 3.15 | < 0.0001 |
| 17 | Solyc05g050350 | Cyclic nucleotide-gated channel | | 0.97 | 0.0558 | 2.89 | < 0.0001 | 3.42 | < 0.0001 | 3.98 | < 0.0001 | 3.23 | < 0.0001 |
| 18 | Solyc12g049030 | Fatty acid desaturase | | 0.33 | 0.9994 | 2.75 | 0.0006 | 3.36 | < 0.0001 | 3.11 | < 0.0001 | 2.98 | 0.0001 |
| 19 | Solyc05g007950 | Ribonuclease LE | | 2.50 | < 0.0001 | 3.41 | < 0.0001 | 3.33 | < 0.0001 | 4.10 | < 0.0001 | 4.16 | < 0.0001 |
| 20 | Solyc01g059965 | β-1,3-glucanase | | 0.34 | 0.9994 | 2.82 | < 0.0001 | 3.33 | < 0.0001 | 3.81 | < 0.0001 | 2.96 | < 0.0001 |
| 21 | Solyc04g074770 | Unknown protein | YES | 1.64 | < 0.0001 | 3.25 | < 0.0001 | 3.26 | < 0.0001 | 2.54 | < 0.0001 | 1.22 | 0.0001 |
| 22 | Solyc03g044830 | Transducin/WD40 repeat-like protein | | 0.28 | 0.9994 | 2.64 | < 0.0001 | 3.23 | < 0.0001 | 3.79 | < 0.0001 | 2.57 | < 0.0001 |
| 23 | Solyc03g020060 | Proteinase inhibitor IId | | 0.91 | 0.6783 | 3.02 | < 0.0001 | 3.17 | < 0.0001 | 1.75 | < 0.0001 | 0.62 | 0.2031 |
| 24 | Solyc10g078230 | Cytochrome P450 | | -0.01 | 0.9994 | 2.66 | < 0.0001 | 3.17 | < 0.0001 | 4.32 | < 0.0001 | 3.16 | < 0.0001 |
| 25 | Solyc02g067750 | Carbonic anhydrase | YES | 1.03 | 0.7322 | 2.76 | < 0.0001 | 3.13 | < 0.0001 | 3.02 | < 0.0001 | 1.65 | 0.0010 |

**Table S4.** List of the 25 most highly up-regulated genes in tomato (*Solanum lycopersicum*) leaves after seven days of infestation with *Tetranychus evansi*. Shown are $Log_2$ fold change ($Log_2FC$) values, representing relative expression levels of tomato genes upon infestation with either a single mite species (*T. urticae* [Tu], *T. evansi* [Te], or *Aculops lycopersici* [Al]) or two species simultaneously (*T. urticae* plus *T. evansi* [Tu+Te], or *T. urticae* plus *A. lycopersici* [Tu+Al]) as compared to non-infested controls, along with their corresponding Benjamini and Hochberg false discovery rate adjusted *P* values (BH-adj *P*). Information in the column with header 'JA' indicates whether expression of the respective gene was found to be significantly induced in the tomato JA-biosynthesis mutant *def-1*, 24 hours after exogenous application of JA (for details see [39]). The asterisk denotes that JA-inducibility of the respective gene was established by [75]. Genes were ranked according to their relative expression level in the Te sample (shaded grey).

| | | | | Te | | Tu+Te | | Tu | | Tu+Al | | Al | |
|---|---|---|---|---|---|---|---|---|---|---|---|---|---|
| **#** | **Locus identifier** | **Annotation (ITAG 3.2)** | **JA** | **$Log_2FC$** | **BH-adj *P*** | **$Log_2FC$** | **BH-adj *P*** | **$Log_2FC$** | **BH-adj *P*** | **$Log_2FC$** | **BH-adj *P*** | **$Log_2FC$** | **BH-adj *P*** |
| 1 | Solyc03g098760 | Kunitz-type trypsin inhibitor | YES | 2.85 | < 0.0001 | 4.64 | < 0.0001 | 4.70 | < 0.0001 | 3.48 | < 0.0001 | 2.16 | < 0.0001 |
| 2 | Solyc05g007950 | Ribonuclease LE | | 2.50 | < 0.0001 | 3.41 | < 0.0001 | 3.33 | < 0.0001 | 4.10 | < 0.0001 | 4.16 | < 0.0001 |
| 3 | Solyc05g014590 | bHLH DNA-binding protein | | 2.19 | < 0.0001 | 3.77 | < 0.0001 | 3.89 | < 0.0001 | 4.49 | < 0.0001 | 3.78 | < 0.0001 |
| 4 | Solyc03g098720 | Kunitz-type trypsin inhibitor | YES | 2.04 | 0.0104 | 3.70 | < 0.0001 | 3.69 | < 0.0001 | 1.59 | 0.0025 | 1.13 | 0.0527 |
| 5 | Solyc10g005320 | Tryptophan synthase | YES | 1.81 | 0.0004 | 4.14 | < 0.0001 | 4.36 | < 0.0001 | 4.31 | < 0.0001 | 2.41 | < 0.0001 |
| 6 | Solyc04g074770 | Unknown protein | YES | 1.64 | < 0.0001 | 3.25 | < 0.0001 | 3.26 | < 0.0001 | 2.54 | < 0.0001 | 1.22 | 0.0001 |
| 7 | Solyc09g091550 | Salicylic acid methyltransferase | YES* | 1.59 | < 0.0001 | 3.58 | < 0.0001 | 3.64 | < 0.0001 | 3.78 | < 0.0001 | 2.87 | < 0.0001 |
| 8 | Solyc12g010020 | Leucine aminopeptidase A1 | YES | 1.58 | 0.0316 | 2.63 | < 0.0001 | 2.17 | < 0.0001 | 1.35 | 0.0024 | 0.70 | 0.1912 |
| 9 | Solyc01g105450 | ABC transporter | YES | 1.55 | 0.0014 | 3.32 | < 0.0001 | 3.79 | < 0.0001 | 3.38 | < 0.0001 | 2.36 | < 0.0001 |
| 10 | Solyc10g075110 | Non-specific lipid-transfer protein | | 1.49 | 0.0003 | 1.64 | < 0.0001 | 0.71 | 0.0354 | 3.21 | < 0.0001 | 3.76 | < 0.0001 |
| 11 | Solyc02g093580 | Pectate lyase | | 1.45 | < 0.0001 | 2.74 | < 0.0001 | 2.71 | < 0.0001 | 3.35 | < 0.0001 | 2.99 | < 0.0001 |
| 12 | Solyc04g079730 | Allene oxide synthase | YES | 1.40 | < 0.0001 | 1.72 | < 0.0001 | 1.67 | < 0.0001 | 1.85 | < 0.0001 | 1.72 | < 0.0001 |
| 13 | Solyc08g036640 | Jasmonate ZIM-domain protein 9 | YES | 1.36 | 0.0004 | 2.15 | < 0.0001 | 2.30 | < 0.0001 | 2.57 | < 0.0001 | 2.50 | < 0.0001 |
| 14 | Solyc01g094250 | Short-chain dehydrogenase/reductase | | 1.35 | 0.0329 | 2.14 | < 0.0001 | 2.12 | < 0.0001 | 2.37 | < 0.0001 | 1.68 | < 0.0001 |
| 15 | Solyc08g074682 | Polyphenol oxidase precursor | | 1.35 | 0.0104 | 3.52 | < 0.0001 | 3.60 | < 0.0001 | 3.61 | < 0.0001 | 0.85 | 0.0240 |
| 16 | Solyc10g078360 | Short-chain dehydrogenase/reductase | YES | 1.34 | < 0.0001 | 2.40 | < 0.0001 | 2.34 | < 0.0001 | 1.60 | < 0.0001 | 1.03 | < 0.0001 |
| 17 | Solyc01g021600 | Dirigent protein | | 1.27 | 0.0027 | 2.86 | < 0.0001 | 2.91 | < 0.0001 | 4.73 | < 0.0001 | 4.63 | < 0.0001 |
| 18 | Solyc02g077420 | Phospholipase A1 | YES | 1.22 | 0.0207 | 2.44 | < 0.0001 | 2.46 | < 0.0001 | 2.37 | < 0.0001 | 2.23 | < 0.0001 |
| 19 | Solyc08g036660 | Jasmonate ZIM-domain protein 11 | | 1.19 | 0.0191 | 2.35 | < 0.0001 | 2.70 | < 0.0001 | 3.41 | < 0.0001 | 2.66 | < 0.0001 |
| 20 | Solyc02g093250 | Caffeoyl-CoA O-methyltransferase | | 1.16 | 0.0160 | 1.63 | < 0.0001 | 1.89 | < 0.0001 | 2.65 | < 0.0001 | 2.75 | < 0.0001 |
| 21 | Solyc01g109710 | Universal stress protein | YES | 1.03 | 0.0017 | 1.90 | < 0.0001 | 1.86 | < 0.0001 | 2.49 | < 0.0001 | 1.81 | < 0.0001 |
| 22 | Solyc06g075690 | Auxin-regulated protein | | 1.02 | 0.0027 | 2.17 | < 0.0001 | 2.24 | < 0.0001 | 3.39 | < 0.0001 | 3.06 | < 0.0001 |
| 23 | Solyc03g013160 | Amino acid transporter, putative | YES | 1.01 | 0.0034 | 2.03 | < 0.0001 | 2.07 | < 0.0001 | 2.01 | < 0.0001 | 1.59 | < 0.0001 |
| 24 | Solyc05g051480 | DNA-directed RNA polymerase subunit | | 1.00 | 0.0473 | 2.73 | < 0.0001 | 2.66 | < 0.0001 | 3.23 | < 0.0001 | 2.89 | < 0.0001 |
| 25 | Solyc08g068680 | Aromatic amino acid decarboxylase 1A | | 1.00 | 0.0034 | 2.84 | < 0.0001 | 3.03 | < 0.0001 | 3.70 | < 0.0001 | 3.01 | < 0.0001 |

**Table S5.** List of the 25 most highly up-regulated genes in tomato (*Solanum lycopersicum*) leaves after seven days of infestation with *Aculops lycopersici*. Shown are $Log_2$ fold change ($Log_2FC$) values, representing relative expression levels of tomato genes upon infestation with either a single mite species (*Tetranychus urticae* [Tu], *T. evansi* [Te], or *A. lycopersici* [Al]) or two species simultaneously (*T. urticae* plus *T. evansi* [Tu+Te], or *T. urticae* plus *A. lycopersici* [Tu+Al]) as compared to non-infested controls, along with their corresponding Benjamini and Hochberg false discovery rate adjusted *P* values (BH-adj *P*). Information in the column with header 'JA' indicates whether expression of the respective gene was found to be significantly induced in the tomato JA-biosynthesis mutant *def-1*, 24 hours after exogenous application of JA (for details see [39]; note that none of the genes in this top list were JA-inducible according to these criteria). Genes were ranked according to their relative expression level in the Al sample (shaded grey).

| | | | | Te | | Tu+Te | | Tu | | Tu+Al | | Al | |
|---|---|---|---|---|---|---|---|---|---|---|---|---|---|
| **#** | **Locus identifier** | **Annotation (ITAG 3.2)** | **JA** | **$Log_2FC$** | **BH-adj *P*** | **$Log_2FC$** | **BH-adj *P*** | **$Log_2FC$** | **BH-adj *P*** | **$Log_2FC$** | **BH-adj *P*** | **$Log_2FC$** | **BH-adj *P*** |
| 1 | Solyc01g021600 | Dirigent protein | | 1.27 | 0.0027 | 2.86 | < 0.0001 | 2.91 | < 0.0001 | 4.73 | < 0.0001 | 4.63 | < 0.0001 |
| 2 | Solyc05g007950 | Ribonuclease LE | | 2.50 | < 0.0001 | 3.41 | < 0.0001 | 3.33 | < 0.0001 | 4.10 | < 0.0001 | 4.16 | < 0.0001 |
| 3 | Solyc02g062890 | Polyol monosaccharide transporter 5 | | 0.02 | 0.9994 | 2.44 | < 0.0001 | 2.77 | < 0.0001 | 4.06 | < 0.0001 | 3.85 | < 0.0001 |
| 4 | Solyc05g014590 | bHLH DNA-binding protein | | 2.19 | < 0.0001 | 3.77 | < 0.0001 | 3.89 | < 0.0001 | 4.49 | < 0.0001 | 3.78 | < 0.0001 |
| 5 | Solyc10g075110 | Non-specific lipid-transfer protein | | 1.49 | 0.0003 | 1.64 | < 0.0001 | 0.71 | 0.0354 | 3.21 | < 0.0001 | 3.76 | < 0.0001 |
| 6 | Solyc01g105070 | Peroxidase precursor | | 0.78 | 0.0539 | 2.12 | < 0.0001 | 2.37 | < 0.0001 | 3.84 | < 0.0001 | 3.75 | < 0.0001 |
| 7 | Solyc02g071130 | WRKY transcription factor 71 | | 0.76 | 0.5048 | 2.39 | < 0.0001 | 2.50 | < 0.0001 | 3.91 | < 0.0001 | 3.43 | < 0.0001 |
| 8 | Solyc08g067630 | Histone deacetylase-like protein | | 1.58 | 0.1823 | 3.31 | < 0.0001 | 3.52 | < 0.0001 | 4.37 | < 0.0001 | 3.39 | < 0.0001 |
| 9 | Solyc05g005865 | Major latex-like protein | | 0.73 | 0.6783 | 1.00 | 0.0078 | 0.34 | 0.4910 | 2.51 | < 0.0001 | 3.36 | < 0.0001 |
| 10 | Solyc04g071070 | Extensin | | 0.96 | 0.5886 | 2.28 | < 0.0001 | 1.83 | < 0.0001 | 3.56 | < 0.0001 | 3.34 | < 0.0001 |
| 11 | Solyc10g083290 | Extracellular invertase LIN6 | | 0.36 | 0.9994 | 1.93 | < 0.0001 | 2.55 | < 0.0001 | 3.62 | < 0.0001 | 3.29 | < 0.0001 |
| 12 | Solyc10g083690 | Cytochrome P450 | | 0.54 | 0.9994 | 3.49 | < 0.0001 | 4.29 | < 0.0001 | 5.30 | < 0.0001 | 3.29 | < 0.0001 |
| 13 | Solyc01g080570 | Inosine-uridine preferring nucleoside hydrolase | | 0.72 | 0.9994 | 3.61 | < 0.0001 | 4.22 | < 0.0001 | 4.65 | < 0.0001 | 3.29 | < 0.0001 |
| 14 | Solyc01g060020 | β-1,3-glucanase | | 0.43 | 0.9994 | 2.90 | < 0.0001 | 3.53 | < 0.0001 | 4.13 | < 0.0001 | 3.26 | < 0.0001 |
| 15 | Solyc05g050350 | Cyclic nucleotide-gated channel | | 0.97 | 0.0558 | 2.89 | < 0.0001 | 3.42 | < 0.0001 | 3.98 | < 0.0001 | 3.23 | < 0.0001 |
| 16 | Solyc01g073820 | Unknown protein | | 0.52 | 0.9743 | 1.98 | < 0.0001 | 2.46 | < 0.0001 | 3.23 | < 0.0001 | 3.22 | < 0.0001 |
| 17 | Solyc10g078230 | Cytochrome P450 | | -0.01 | 0.9994 | 2.66 | < 0.0001 | 3.17 | < 0.0001 | 4.32 | < 0.0001 | 3.16 | < 0.0001 |
| 18 | Solyc01g059980 | β-1,3-glucanase | | 0.36 | 0.9994 | 3.03 | < 0.0001 | 3.45 | < 0.0001 | 3.96 | < 0.0001 | 3.15 | < 0.0001 |
| 19 | Solyc01g059990 | Serine/threonine protein phosphatase | | 0.34 | 0.9994 | 3.03 | < 0.0001 | 3.51 | < 0.0001 | 3.94 | < 0.0001 | 3.15 | < 0.0001 |
| 20 | Solyc04g072000 | Chitinase | | 0.65 | 0.5372 | 2.89 | < 0.0001 | 3.00 | < 0.0001 | 3.89 | < 0.0001 | 3.13 | < 0.0001 |
| 21 | Solyc02g036480 | Unknown protein | | 0.77 | 0.3696 | 2.59 | < 0.0001 | 2.83 | < 0.0001 | 3.56 | < 0.0001 | 3.09 | < 0.0001 |
| 22 | Solyc01g006950 | Syntaxin | | 0.85 | 0.3007 | 2.71 | < 0.0001 | 2.84 | < 0.0001 | 3.74 | < 0.0001 | 3.07 | < 0.0001 |
| 23 | Solyc06g075690 | Auxin-regulated protein | | 1.02 | 0.0027 | 2.17 | < 0.0001 | 2.24 | < 0.0001 | 3.39 | < 0.0001 | 3.06 | < 0.0001 |
| 24 | Solyc09g009985 | Unknown protein | | 0.57 | 0.7793 | 1.87 | < 0.0001 | 2.19 | < 0.0001 | 3.37 | < 0.0001 | 3.04 | < 0.0001 |
| 25 | Solyc04g011750 | Unknown protein | | 0.18 | 0.9994 | 1.52 | 0.0002 | 1.80 | < 0.0001 | 3.32 | < 0.0001 | 3.02 | < 0.0001 |

**Table S6.** List of the 25 most strongly down-regulated genes in tomato (*Solanum lycopersicum*) leaves after seven days of infestation with *Aculops lycopersici*. Shown are $Log_2$ fold change ($Log_2FC$) values, representing relative expression levels of tomato genes upon infestation with either a single mite species (*Tetranychus urticae* [Tu], *T. evansi* [Te], or *A. lycopersici* [Al]) or two species simultaneously (*T. urticae* plus *T. evansi* [Tu+Te], or *T. urticae* plus *A. lycopersici* [Tu+Al]) as compared to non-infested controls, along with their corresponding Benjamini and Hochberg false discovery rate adjusted *P* values (BH-adj *P*). Information in the column with header 'JA' indicates whether expression of the respective gene was found to be significantly induced in the tomato JA-biosynthesis mutant *def-1*, 24 hours after exogenous application of JA (for details see [39]); note that none of the genes in this top list were JA-inducible according to these criteria. Genes were ranked according to their relative expression level in the Al sample (shaded grey).

| | | | | Te | | Tu+Te | | Tu | | Tu+Al | | Al | |
|---|---|---|---|---|---|---|---|---|---|---|---|---|---|
| # | Locus identifier | Annotation (ITAG 3.2) | JA | $Log_2FC$ | BH-adj *P* | $Log_2FC$ | BH-adj *P* | $Log_2FC$ | BH-adj *P* | $Log_2FC$ | BH-adj *P* | $Log_2FC$ | BH-adj *P* |
| 1 | Solyc11g056680 | LRR receptor-like kinase | | -0.63 | 0.9994 | -1.00 | 0.0387 | -1.57 | 0.0004 | -3.03 | < 0.0001 | -2.39 | < 0.0001 |
| 2 | Solyc07g063905 | RING/U-box protein | | -0.23 | 0.9994 | -0.27 | 0.7215 | -0.55 | 0.3009 | -2.32 | < 0.0001 | -2.08 | < 0.0001 |
| 3 | Solyc08g005680 | Cis-prenyltransferase 1 / Neryl diphosphate synthase 1 | | -0.26 | 0.9994 | -0.07 | 0.9190 | -0.61 | 0.0948 | -2.35 | < 0.0001 | -1.97 | < 0.0001 |
| 4 | Solyc03g120380 | IAA19 | | -0.92 | 0.1316 | -0.85 | 0.0148 | -1.35 | < 0.0001 | -2.15 | < 0.0001 | -1.94 | < 0.0001 |
| 5 | Solyc10g008720 | GDSL esterase/lipase | | -0.31 | 0.9994 | -0.78 | 0.1329 | -0.93 | 0.0508 | -2.72 | < 0.0001 | -1.93 | < 0.0001 |
| 6 | Solyc06g069730 | Chlorophyll A-B binding protein | | -0.60 | 0.9994 | -1.18 | 0.0391 | -1.27 | 0.0177 | -2.18 | < 0.0001 | -1.93 | 0.0002 |
| 7 | Solyc08g067030 | Unknown protein | | -0.50 | 0.9994 | -0.45 | 0.6107 | -1.11 | 0.0622 | -2.32 | < 0.0001 | -1.86 | 0.0008 |
| 8 | Solyc07g018240 | Long-chain fatty acid elongase | | -0.42 | 0.9994 | -0.90 | 0.0055 | -1.16 | 0.0002 | -2.39 | < 0.0001 | -1.77 | < 0.0001 |
| 9 | Solyc06g071320 | Unknown protein | | -0.48 | 0.9994 | -1.43 | 0.0004 | -1.60 | < 0.0001 | -2.49 | < 0.0001 | -1.75 | < 0.0001 |
| 10 | Solyc08g014430 | Formin 3 (actin-binding) | | -0.21 | 0.9994 | -0.84 | 0.0313 | -0.78 | 0.0390 | -1.99 | < 0.0001 | -1.75 | < 0.0001 |
| 11 | Solyc03g116730 | Stearoyl-CoA 9-desaturase | | -0.30 | 0.9994 | -0.93 | 0.0146 | -0.83 | 0.0234 | -1.89 | < 0.0001 | -1.70 | < 0.0001 |
| 12 | Solyc12g055970 | Endoglucanase | | -0.13 | 0.9994 | -0.60 | 0.1126 | -0.61 | 0.0828 | -1.58 | < 0.0001 | -1.64 | < 0.0001 |
| 13 | Solyc12g011010 | Meiosis 5 | | -0.42 | 0.9994 | -0.96 | 0.0918 | -1.83 | 0.0003 | -2.72 | < 0.0001 | -1.64 | 0.0009 |
| 14 | Solyc09g082660 | Caffeoyl-CoA O-methyltransferase | | -0.15 | 0.9994 | -1.43 | 0.0001 | -1.27 | 0.0003 | -2.84 | < 0.0001 | -1.63 | < 0.0001 |
| 15 | Solyc10g083670 | Glycosyltransferase | | -0.23 | 0.9994 | -0.26 | 0.7009 | -0.75 | 0.0796 | -1.84 | < 0.0001 | -1.62 | < 0.0001 |
| 16 | Solyc06g009190 | Pectinesterase | | -0.35 | 0.9994 | -1.01 | 0.0279 | -1.24 | 0.0036 | -2.74 | < 0.0001 | -1.62 | 0.0001 |
| 17 | Solyc03g098480 | Unknown protein | | 0.04 | 0.9994 | -1.00 | 0.0023 | -0.79 | 0.0138 | -2.31 | < 0.0001 | -1.61 | < 0.0001 |
| 18 | Solyc05g053550 | Chalcone synthase 2 | | -0.12 | 0.9994 | -0.62 | 0.1086 | -0.99 | 0.0036 | -2.36 | < 0.0001 | -1.59 | < 0.0001 |
| 19 | Solyc02g080200 | Pectinesterase | | -0.68 | 0.9994 | -1.04 | 0.0300 | -1.07 | 0.0184 | -2.03 | < 0.0001 | -1.59 | 0.0003 |
| 20 | Solyc10g083300 | Extracellular invertase LIN8 | | -0.46 | 0.9994 | -1.17 | 0.0014 | -1.00 | 0.0049 | -2.12 | < 0.0001 | -1.56 | < 0.0001 |
| 21 | Solyc08g076820 | bHLH transcription factor 146 | | -0.49 | 0.9994 | -1.23 | 0.0003 | -1.46 | < 0.0001 | -2.03 | < 0.0001 | -1.56 | < 0.0001 |
| 22 | Solyc01g006330 | Plastid-lipid-associated protein | | -0.49 | 0.9994 | -1.23 | 0.0002 | -1.25 | 0.0001 | -2.01 | < 0.0001 | -1.56 | < 0.0001 |
| 23 | Solyc12g044280 | Photosystem I reaction center subunit | | -0.21 | 0.9994 | -0.86 | 0.0465 | -0.96 | 0.0169 | -1.79 | < 0.0001 | -1.54 | 0.0001 |
| 24 | Solyc01g110570 | Small auxin up-regulated RNA 4 | | -0.72 | 0.9994 | -1.22 | 0.0240 | -1.33 | 0.0091 | -2.35 | < 0.0001 | -1.54 | 0.0017 |
| 25 | Solyc05g009270 | Long-chain fatty acid elongase | | -0.37 | 0.9994 | -0.54 | 0.0493 | -0.69 | 0.0063 | -1.68 | < 0.0001 | -1.53 | < 0.0001 |

**Table S7.** List of the 25 tomato (*Solanum lycopersicum*) genes that are most highly up-regulated in leaves after seven days of infestation with *Aculops lycopersici* while not differentially regulated after infestation with *Tetranychus urticae*. Shown are $Log_2$ fold change ($Log_2FC$) values, representing relative expression levels of tomato genes upon infestation with either a single mite species (*T. urticae* [Tu], *T. evansi* [Te], or *A. lycopersici* [Al]) or two species simultaneously (*T. urticae* plus *T. evansi* [Tu+Te], or *T. urticae* plus *A. lycopersici* [Tu+Al]) as compared to non-infested controls, along with their corresponding Benjamini and Hochberg false discovery rate adjusted *P* values (BH-adj *P*). Differentially expressed genes were identified based on a BH-adj $P \leq 0.05$. Information in the column with header 'JA' indicates whether expression of the respective gene was found to be significantly induced in the tomato JA-biosynthesis mutant *def-1*, 24 hours after exogenous application of JA (for details see [39]). Genes were ranked according to their relative expression level in the Al sample (shaded grey).

| | | | | Te | | Tu+Te | | Tu | | Tu+Al | | Al | |
|---|---|---|---|---|---|---|---|---|---|---|---|---|---|
| # | Locus identifier | Annotation (ITAG 3.2) | JA | $Log_2FC$ | BH-adj *P* | $Log_2FC$ | BH-adj *P* | $Log_2FC$ | BH-adj *P* | $Log_2FC$ | BH-adj *P* | $Log_2FC$ | BH-adj *P* |
| 1 | Solyc05g005865 | Major latex-like protein | | 0.73 | 0.6783 | 1.00 | 0.0078 | 0.34 | 0.4910 | 2.51 | < 0.0001 | 3.36 | < 0.0001 |
| 2 | Solyc02g071620 | GDSL-motif lipase/hydrolase | | 0.37 | 0.9994 | 0.27 | 0.6431 | 0.52 | 0.2246 | 2.14 | < 0.0001 | 2.43 | < 0.0001 |
| 3 | Solyc09g018020 | Expansin | | 0.04 | 0.9994 | 0.14 | 0.8012 | -0.05 | 0.9338 | 2.13 | < 0.0001 | 2.42 | < 0.0001 |
| 4 | Solyc09g063060 | GDSL esterase/lipase | | 0.09 | 0.9994 | 0.26 | 0.5577 | 0.19 | 0.7104 | 1.77 | < 0.0001 | 2.25 | < 0.0001 |
| 5 | Solyc06g061280 | Cinnamoyl-CoA reductase | | 0.20 | 0.9994 | 0.71 | 0.0529 | 0.56 | 0.1252 | 2.24 | < 0.0001 | 2.22 | < 0.0001 |
| 6 | Solyc01g011430 | O-acyltransferase | | 0.22 | 0.9994 | 0.29 | 0.5379 | 0.23 | 0.6339 | 1.39 | < 0.0001 | 1.95 | < 0.0001 |
| 7 | Solyc01g105710 | Peptidyl-prolyl isomerase | | 0.43 | 0.6890 | 0.21 | 0.4765 | -0.28 | 0.2811 | 1.34 | < 0.0001 | 1.90 | < 0.0001 |
| 8 | Solyc01g079940 | Xylanase inhibitor | | 0.22 | 0.9994 | 0.36 | 0.2379 | 0.44 | 0.0961 | 1.78 | < 0.0001 | 1.90 | < 0.0001 |
| 9 | Solyc07g052135 | Sesquiterpene synthase | | -0.17 | 0.9994 | 1.24 | 0.1438 | 1.46 | 0.0576 | 2.42 | 0.0003 | 1.88 | 0.0085 |
| 10 | Solyc11g067190 | Fatty acyl-CoA reductase | | 0.01 | 0.9994 | 0.54 | 0.4487 | 0.79 | 0.1684 | 2.72 | < 0.0001 | 1.86 | 0.0002 |
| 11 | Solyc12g011030 | Xyloglucan endotransglucosylase-hydrolase | | 0.02 | 0.9994 | 0.73 | 0.0868 | 0.73 | 0.0667 | 1.71 | < 0.0001 | 1.82 | < 0.0001 |
| 12 | Solyc05g009430 | Endonuclease | | 0.19 | 0.9994 | 0.50 | 0.1407 | 0.57 | 0.0643 | 1.81 | < 0.0001 | 1.81 | < 0.0001 |
| 13 | Solyc03g082690 | U-box domain-containing protein | | 0.31 | 0.9994 | 0.68 | 0.3305 | 1.04 | 0.0672 | 1.54 | 0.0017 | 1.66 | 0.0015 |
| 14 | Solyc01g098590 | Phosphoglycerate mutase | | 0.50 | 0.9994 | 0.82 | 0.4476 | 0.99 | 0.3009 | 1.30 | 0.0801 | 1.61 | 0.0401 |
| 15 | Solyc02g070890 | LRR receptor like kinase FLS2 | | 0.06 | 0.9994 | 0.46 | 0.3107 | 0.62 | 0.1103 | 1.46 | < 0.0001 | 1.60 | < 0.0001 |
| 16 | Solyc09g097770 | Glycine-rich cell wall protein | | 0.67 | 0.9994 | 1.32 | 0.1063 | 1.28 | 0.1020 | 1.79 | 0.0069 | 1.53 | 0.0347 |
| 17 | Solyc05g055400 | Cytochrome P450 | | 0.04 | 0.9994 | 0.43 | 0.4237 | 0.12 | 0.8797 | 0.95 | 0.0074 | 1.49 | 0.0001 |
| 18 | Solyc06g065820 | Ethylene Response Factor H.1 | | -0.17 | 0.9994 | 0.56 | 0.0485 | 0.35 | 0.2647 | 1.44 | < 0.0001 | 1.47 | < 0.0001 |
| 19 | Solyc07g049440 | GDSL esterase/lipase | | 0.30 | 0.9994 | 0.14 | 0.8380 | -0.36 | 0.4600 | 0.54 | 0.1168 | 1.45 | < 0.0001 |
| 20 | Solyc06g007460 | Epidermal patterning factor | | 0.12 | 0.9994 | 0.90 | 0.0038 | 0.57 | 0.0738 | 1.61 | < 0.0001 | 1.44 | < 0.0001 |
| 21 | Solyc06g074790 | Histone H2B | YES | 0.18 | 0.9994 | 0.71 | 0.3261 | 0.74 | 0.2685 | 1.15 | 0.0273 | 1.41 | 0.0102 |
| 22 | Solyc03g093120 | Xyloglucan endotransglucosylase-hydrolase | | 0.26 | 0.9994 | 0.84 | 0.0356 | 0.76 | 0.0512 | 1.39 | 0.0001 | 1.39 | 0.0001 |
| 23 | Solyc05g055080 | Unknown protein | | 0.27 | 0.9994 | 0.60 | 0.1773 | 0.63 | 0.1241 | 1.36 | 0.0001 | 1.33 | 0.0003 |
| 24 | Solyc04g050440 | Ammonium transporter | | 0.23 | 0.9994 | 0.39 | 0.2389 | 0.42 | 0.1671 | 1.29 | < 0.0001 | 1.33 | < 0.0001 |
| 25 | Solyc01g010390 | β-glucosidase | | 0.22 | 0.9994 | 0.70 | 0.1031 | 0.54 | 0.2225 | 1.18 | 0.0007 | 1.31 | 0.0004 |

**Table S8.** List of the 25 tomato (*Solanum lycopersicum*) genes that are most strongly down-regulated in leaves after seven days of infestation with *Aculops lycopersici* while not differentially regulated after infestation with *Tetranychus urticae*. Shown are $Log_2$ fold change ($Log_2FC$) values, representing relative expression levels of tomato genes upon infestation with either a single mite species (*T. urticae* [Tu], *T. evansi* [Te], or *A. lycopersici* [Al]) or two species simultaneously (*T. urticae* plus *T. evansi* [Tu+Te], or *T. urticae* plus *A. lycopersici* [Tu+Al]) as compared to non-infested controls, along with their corresponding Benjamini and Hochberg false discovery rate adjusted *P* values (BH-adj *P*). Differentially expressed genes were identified based on a BH-adj *P* ≤ 0.05. Information in the column with header 'JA' indicates whether expression of the respective gene was found to be significantly induced in the tomato JA-biosynthesis mutant *def-1*, 24 hours after exogenous application of JA (for details see [39]). Genes were ranked according to their relative expression level in the Al sample (shaded grey).

| | | | | Te | | Tu+Te | | Tu | | Tu+Al | | Al | |
|---|---|---|---|---|---|---|---|---|---|---|---|---|---|
| **#** | **Locus identifier** | **Annotation (ITAG 3.2)** | **JA** | **$Log_2FC$** | **BH-adj *P*** | **$Log_2FC$** | **BH-adj *P*** | **$Log_2FC$** | **BH-adj *P*** | **$Log_2FC$** | **BH-adj *P*** | **$Log_2FC$** | **BH-adj *P*** |
| 1 | Solyc07g063905 | RING/U-box protein | | -0.23 | 0.9994 | -0.27 | 0.7215 | -0.55 | 0.3009 | -2.32 | < 0.0001 | -2.08 | < 0.0001 |
| 2 | Solyc08g005680 | Cis-prenyltransferase 1 / Neryl diphosphate synthase 1 | | -0.26 | 0.9994 | -0.07 | 0.9190 | -0.61 | 0.0948 | -2.35 | < 0.0001 | -1.97 | < 0.0001 |
| 3 | Solyc10g008720 | GDSL esterase/lipase | | -0.31 | 0.9994 | -0.78 | 0.1329 | -0.93 | 0.0508 | -2.72 | < 0.0001 | -1.93 | < 0.0001 |
| 4 | Solyc08g067030 | Unknown protein | | -0.50 | 0.9994 | -0.45 | 0.6107 | -1.11 | 0.0622 | -2.32 | < 0.0001 | -1.86 | 0.0008 |
| 5 | Solyc12g055970 | Endoglucanase | | -0.13 | 0.9994 | -0.60 | 0.1126 | -0.61 | 0.0828 | -1.58 | < 0.0001 | -1.64 | < 0.0001 |
| 6 | Solyc10g083670 | Glycosyltransferase | | -0.23 | 0.9994 | -0.26 | 0.7009 | -0.75 | 0.0796 | -1.84 | < 0.0001 | -1.62 | < 0.0001 |
| 7 | Solyc06g066530 | Unknown protein | | 0.01 | 0.9994 | -0.30 | 0.5819 | -0.50 | 0.2325 | -2.14 | < 0.0001 | -1.50 | < 0.0001 |
| 8 | Solyc04g007825 | MLP-like protein | | -0.28 | 0.9994 | -0.17 | 0.7947 | -0.38 | 0.3891 | -1.69 | < 0.0001 | -1.47 | < 0.0001 |
| 9 | Solyc06g084050 | Photosystem II reaction center subunit | | -0.24 | 0.9994 | -0.57 | 0.4402 | -0.93 | 0.1103 | -1.95 | 0.0001 | -1.41 | 0.0073 |
| 10 | Solyc04g050820 | Inositol-3-phosphate synthase | | -0.28 | 0.9994 | -0.45 | 0.5232 | -0.65 | 0.2673 | -1.87 | < 0.0001 | -1.35 | 0.0044 |
| 11 | Solyc01g108630 | Nitrite reductase | | -0.34 | 0.9994 | -0.32 | 0.5939 | -0.26 | 0.6997 | -1.34 | 0.0003 | -1.34 | 0.0006 |
| 12 | Solyc04g071340 | Fructose-1,6-bisphosphatase | | -0.27 | 0.9994 | -0.80 | 0.0811 | -0.67 | 0.1434 | -1.57 | < 0.0001 | -1.31 | 0.0011 |
| 13 | Solyc02g091920 | Xyloglucan endotransglucosylase-hydrolase | | -0.24 | 0.9994 | -0.62 | 0.3978 | -0.02 | 0.9863 | -1.20 | 0.0176 | -1.30 | 0.0156 |
| 14 | Solyc06g064550 | Aspartokinase-homoserine dehydrogenase | | -0.20 | 0.9994 | -0.81 | 0.0368 | -0.64 | 0.0950 | -1.49 | < 0.0001 | -1.30 | 0.0002 |
| 15 | Solyc10g078530 | Patatin (lipid acyl hydrolase) | | -0.22 | 0.9994 | 0.28 | 0.4569 | -0.20 | 0.6326 | -1.45 | < 0.0001 | -1.29 | < 0.0001 |
| 16 | Solyc01g087040 | Thylakoid luminal protein | | -0.28 | 0.9994 | -1.00 | 0.0237 | -0.84 | 0.0509 | -1.68 | < 0.0001 | -1.27 | 0.0015 |
| 17 | Solyc06g072710 | RNA polymerase sigma factor | | -0.16 | 0.9994 | -0.69 | 0.1283 | -0.74 | 0.0780 | -1.47 | 0.0001 | -1.26 | 0.0010 |
| 18 | Solyc06g060830 | Homeobox-leucine zipper protein | | -0.28 | 0.9994 | -0.47 | 0.3721 | -0.31 | 0.6067 | -1.01 | 0.0060 | -1.26 | 0.0012 |
| 19 | Solyc12g099650 | Photosystem II subunit | | -0.01 | 0.9994 | -0.20 | 0.8556 | -0.49 | 0.5258 | -1.81 | 0.0003 | -1.26 | 0.0175 |
| 20 | Solyc06g053840 | IAA4 | | -0.34 | 0.9994 | -0.41 | 0.4022 | -0.55 | 0.1793 | -1.51 | < 0.0001 | -1.26 | 0.0004 |
| 21 | Solyc06g062370 | Acid phosphatase | YES | -0.10 | 0.9994 | 1.35 | 0.0180 | 0.62 | 0.3598 | -0.97 | 0.0536 | -1.24 | 0.0189 |
| 22 | Solyc03g006410 | Unknown protein | | -0.53 | 0.9994 | -0.83 | 0.2320 | -0.81 | 0.2161 | -1.73 | 0.0008 | -1.24 | 0.0271 |
| 23 | Solyc10g054870 | Triosephosphate isomerase | | -0.64 | 0.9994 | -0.48 | 0.4413 | -0.89 | 0.0587 | -1.58 | 0.0001 | -1.24 | 0.0046 |
| 24 | Solyc12g011280 | Chlorophyll A-B binding protein | | -0.27 | 0.9994 | -0.87 | 0.0519 | -0.82 | 0.0549 | -1.76 | < 0.0001 | -1.23 | 0.0020 |
| 25 | Solyc10g054380 | Fructose-bisphosphate aldolase | | -0.42 | 0.9994 | -0.70 | 0.1008 | -0.49 | 0.2920 | -1.53 | < 0.0001 | -1.22 | 0.0010 |

**Table S9.** List of the 25 most strongly down-regulated genes in tomato (*Solanum lycopersicum*) leaves after seven days of infestation with *Tetranychus urticae*. Shown are $Log_2$ fold change ($Log_2FC$) values, representing relative expression levels of tomato genes upon infestation with either a single mite species (*T. urticae* [Tu], *T. evansi* [Te], or *Aculops lycopersici* [Al]) or two species simultaneously (*T. urticae* plus *T. evansi* [Tu+Te], or *T. urticae* plus *A. lycopersici* [Tu+Al]) as compared to non-infested controls, along with their corresponding Benjamini and Hochberg false discovery rate adjusted *P* values (BH-adj *P*). Information in the column with header 'JA' indicates whether expression of the respective gene was found to be significantly induced in the tomato JA-biosynthesis mutant *def-1*, 24 hours after exogenous application of JA (for details see [39]; note that none of the genes in this top list were JA-inducible according to these criteria. Genes were ranked according to their relative expression level in the Tu sample (shaded grey).

| | | | | Te | | Tu+Te | | Tu | | Tu+Al | | Al | |
|---|---|---|---|---|---|---|---|---|---|---|---|---|---|
| **#** | **Locus identifier** | **Annotation (ITAG 3.2)** | **JA** | **$Log_2FC$** | **BH-adj *P*** | **$Log_2FC$** | **BH-adj *P*** | **$Log_2FC$** | **BH-adj *P*** | **$Log_2FC$** | **BH-adj *P*** | **$Log_2FC$** | **BH-adj *P*** |
| 1 | Solyc11g010410 | 2-oxoglutarate (2OG) and Fe(II)-dependent oxygenase | | -0.74 | 0.9994 | -1.81 | 0.0001 | -1.84 | 0.0001 | -2.17 | < 0.0001 | -1.51 | 0.0006 |
| 2 | Solyc12g011010 | Meiosis 5 | | -0.42 | 0.9994 | -0.96 | 0.0918 | -1.83 | 0.0003 | -2.72 | < 0.0001 | -1.64 | 0.0009 |
| 3 | Solyc06g068130 | Tetratricopeptide repeat protein | | 0.39 | 0.9994 | -1.68 | 0.0153 | -1.75 | 0.0077 | -1.93 | 0.0012 | -0.48 | 0.5980 |
| 4 | Solyc12g009650 | Lipid-transfer protein, putative | | -0.61 | 0.9994 | -1.52 | 0.0078 | -1.75 | 0.0013 | -2.28 | < 0.0001 | -1.44 | 0.0066 |
| 5 | Solyc09g008320 | Xyloglucan endotrans-glucosylase/hydrolase | | -0.38 | 0.9994 | -1.26 | 0.0035 | -1.75 | < 0.0001 | -2.42 | < 0.0001 | -1.34 | 0.0009 |
| 6 | Solyc10g009150 | Organ-specific protein S2 | | -0.90 | 0.9994 | -0.90 | 0.3200 | -1.72 | 0.0159 | -1.57 | 0.0151 | -1.06 | 0.1590 |
| 7 | Solyc00g272810 | N-acetyltransferase | | -0.85 | 0.9994 | -0.94 | 0.1176 | -1.68 | 0.0014 | -1.38 | 0.0042 | -0.42 | 0.5598 |
| 8 | Solyc06g071320 | Unknown protein | | -0.48 | 0.9994 | -1.43 | 0.0004 | -1.60 | < 0.0001 | -2.49 | < 0.0001 | -1.75 | < 0.0001 |
| 9 | Solyc11g056680 | LRR receptor-like kinase | | -0.63 | 0.9994 | -1.00 | 0.0387 | -1.57 | 0.0004 | -3.03 | < 0.0001 | -2.39 | < 0.0001 |
| 10 | Solyc07g041720 | Oxalate oxidase-like germin protein | | -0.50 | 0.9994 | -1.61 | 0.0015 | -1.53 | 0.0018 | -2.16 | < 0.0001 | -0.96 | 0.0534 |
| 11 | Solyc01g089850 | Cyclin-dependent protein kinase | | -0.73 | 0.3304 | -1.50 | < 0.0001 | -1.52 | < 0.0001 | -2.03 | < 0.0001 | -0.98 | 0.0007 |
| 12 | Solyc10g074540 | Unknown protein | | -0.86 | 0.8643 | -1.60 | 0.0008 | -1.51 | 0.0010 | -1.42 | 0.0008 | -1.23 | 0.0061 |
| 13 | Solyc08g063090 | Delta-6-desaturase | | -0.41 | 0.9994 | -1.59 | 0.0001 | -1.49 | 0.0002 | -2.16 | < 0.0001 | -0.97 | 0.0144 |
| 14 | Solyc03g112540 | Short-chain dehydrogenase/reductase | | -0.46 | 0.9994 | -1.44 | 0.0005 | -1.48 | 0.0002 | -1.86 | < 0.0001 | -1.10 | 0.0046 |
| 15 | Solyc02g076690 | Cysteine protease | | -0.77 | 0.9994 | -1.56 | 0.0028 | -1.47 | 0.0034 | -2.15 | < 0.0001 | -1.04 | 0.0391 |
| 16 | Solyc01g005300 | F box protein | | -0.88 | 0.9994 | -1.32 | 0.0582 | -1.47 | 0.0249 | -1.39 | 0.0185 | -0.61 | 0.4502 |
| 17 | Solyc08g076820 | bHLH transcription factor 146 | | -0.49 | 0.9994 | -1.23 | 0.0003 | -1.46 | < 0.0001 | -2.03 | < 0.0001 | -1.56 | < 0.0001 |
| 18 | Solyc07g052980 | Xyloglucan endotrans-glycosylase/hydrolase | | -0.57 | 0.9994 | -1.23 | 0.0003 | -1.44 | < 0.0001 | -2.11 | < 0.0001 | -1.07 | 0.0009 |
| 19 | Solyc03g058160 | Zinc finger transcription factor | | -0.51 | 0.9994 | -1.20 | 0.0007 | -1.37 | 0.0001 | -1.73 | < 0.0001 | -0.59 | 0.0972 |
| 20 | Solyc03g120380 | IAA19 | | -0.92 | 0.1316 | -0.85 | 0.0148 | -1.35 | < 0.0001 | -2.15 | < 0.0001 | -1.94 | < 0.0001 |
| 21 | Solyc01g110570 | Small auxin up-regulated RNA 4 | | -0.72 | 0.9994 | -1.22 | 0.0240 | -1.33 | 0.0091 | -2.35 | < 0.0001 | -1.54 | 0.0017 |
| 22 | Solyc05g026490 | Phosphomannomutase/phosphor-glucomutase | | -0.44 | 0.9994 | -1.33 | 0.0002 | -1.32 | 0.0001 | -1.63 | < 0.0001 | -1.04 | 0.0017 |
| 23 | Solyc01g099630 | Xyloglucan endotrans-glycosylase/hydrolase | | -0.57 | 0.9994 | -1.04 | 0.0024 | -1.32 | 0.0001 | -0.95 | 0.0016 | -0.33 | 0.4166 |
| 24 | Solyc02g085020 | Dihydroflavonol 4-reductase | | -0.17 | 0.9994 | -1.21 | 0.0034 | -1.30 | 0.0010 | -1.98 | < 0.0001 | -1.26 | 0.0011 |
| 25 | Solyc07g053540 | Fasciclin-like arabinogalactan protein | | -0.71 | 0.9994 | -1.30 | 0.0037 | -1.30 | 0.0026 | -1.47 | 0.0002 | -0.97 | 0.0229 |

**Table S10.** qPCR primer specifications

| Target Gene | Name | Gene Identifier | Forward Primer (5' → 3') | Reverse Primer (5' → 3') | References |
|---|---|---|---|---|---|
| *JIP-21* | *Jasmonate-inducible protein 21* | Solyc03g098790 | ACTCGTCCTGTGCTTTGTCC | CCCAAGAGGATTTTCGTTGA | Lisón et al. [61] |
| *PI-IIc* | *Proteinase inhibitor IIc* | Solyc03g020050 | CAGGATGTACGACGTGTTGC | GAGTTTGCAACCCTCTCCTG | Gadea et al. [147] |
| *KTI_20* | *Kunitz-type trypsin inhibitor* | Solyc03g098720 | CTTGGGAATCCGACTGTTTG | GCCTGCTTTACCGTTTGTTG | This study |
| *KTI_40* | *Kunitz-type trypsin inhibitor* | Solyc03g098740 | CGTCCGGGGTACACCTACTA | TTTCGGGCTTGAAAAACATC | This study |
| *LapA1* | *Leucine aminopeptidase A1* | Solyc12g010020 | TGATACTCTTGGTCTTACTAGGC | GACCAACTAGAGTAATCCTTCCA | Chao et al. [148] |
| *CYP450* | *Cytochrome P450* | Solyc10g083690 | ATATGGATGAAAGCATGGGAGT | TGGGAAAACAATGTTATGTATCG | This study |
| *Ser PI* | *Serine proteinase inhibitor* | Solyc09g084480 | GGGTGGCTTAATATAATGGAT | GGAATTAATTAATGAGAGAACATA | This study |
| *ICS* | *Isochorismate synthase* | Solyc06g071030 | AACTTGAGGCACCTCTTTTGA | CCCAAAATTGCATATGACAGG | This study |
| *β-1-3-Gluc* | *β-1-3-glucanase* | Solyc01g060020 | TGCAACACCAAATAAATTCGTA | TTTGCAGTTACCAGATCAGAGTG | This study |
| *PR-P* | *Pathogenesis-related protein* | Solyc04g064880 | GCGTCAACATGTCCTTTTATGA | AAATTTGCGTAAACAGAATTACAA | This study |
| *CHS1* | *Chalcone synthase 1* | Solyc09g091510 | TCCTTCACAGTGTTGCTGCT | TAGCAAACACATTAATAACCAAGAAA | O'Neill et al. [88] |
| *CHS2* | *Chalcone synthase 2* | Solyc05g053550 | GGCTCCCACTCGAGACATAA | CCACAAATCTAATTTCCAAATTCTT | O'Neill et al. [88] |
| *GAME1* | *Glycoalkaloid metabolism 1* | Solyc07g043490 | GCATTTTGGTCCGCTCTCTC | GCGCATTCAACCAATCTACAAC | Itkin et al. [107] |
| *GAME9/JRE4* | *Glycoalkaloid metabolism 9 / Jasmonate-responsive ethylene response factor 4* | Solyc01g090340 | TGTTTCCTCCGGTGTTACGG | CGATTTTTTCGAAACTCTTTCC | Cárdenas et al. [113] Thagun et al. [114] |
| *Actin* | *Actin* | Solyc03g078400 | TCAGCACATTCCAGCAGATGT | AACAGACAGGACACTCGCACT | Schimmel et al. [41] |
